# Revising the mechanism of p75NTR activation: intrinsically monomeric state of death domains invokes the "helper" hypothesis

**DOI:** 10.1038/s41598-020-70721-8

**Published:** 2020-08-13

**Authors:** Sergey A. Goncharuk, Lilya E. Artemieva, Kirill D. Nadezhdin, Alexander S. Arseniev, Konstantin S. Mineev

**Affiliations:** 1 Laboratory of Biomolecular NMR Spectroscopy, Shemyakin-Ovchinnikov Institute of Bioorganic Chemistry of the Russian Academy of Sciences, Moscow, Russia; 2 Phystech School of Biological and Medical Physics, Moscow Institute of Physics and Technology, Dolgoprudny, Russia

**Keywords:** Membrane proteins, Solution-state NMR, Neurotrophic factors, Solution-state NMR, Membrane proteins, Mechanism of action, Post-translational modifications

## Abstract

The neurotrophin receptor p75NTR plays crucial roles in neuron development and regulates important neuronal processes like degeneration, apoptosis and cell survival. At the same time the detailed mechanism of signal transduction is unclear. One of the main hypotheses known as the snail-tong mechanism assumes that in the inactive state, the death domains interact with each other and in response to ligand binding there is a conformational change leading to their exposure. Here, we show that neither rat nor human p75NTR death domains homodimerize in solution. Moreover, there is no interaction between the death domains in a more native context: the dimerization of transmembrane domains in liposomes and the presence of activating mutation in extracellular juxtamembrane region do not lead to intracellular domain interaction. These findings suggest that the activation mechanism of p75NTR should be revised. Thus, we propose a novel model of p75NTR functioning based on interaction with “helper” protein.

## Introduction

Neurotrophic factors (NTs) play a key role in neuron functioning, including cell differentiation, migration, apoptosis, survival, neurite outgrowth and synaptic plasticity^1–3^. There are two distinct types of NT receptors: Trk receptor tyrosine kinase family, with each member binding a specific neurotrophin, and p75 neurotrophin receptor (p75NTR), common for all the NTs. Depending on the cell context, p75NTR activation may result in various and sometimes opposite effects such as cell survival or apoptosis^4–8^. It is noteworthy that the expression of p75NTR is downregulated in adults but it can be re-expressed upon different pathological conditions, including neuronal insult, neurodegeneration, axotomy^3,9^. The important role and wide range of p75NTR activities make this receptor a prospective target for structural investigations and drug design.

p75NTR is a type I transmembrane protein and is composed of an extracellular ligand-binding domain (ECD), a single-span helical transmembrane domain (TMD), and an intracellular domain (ICD). The ICD of p75NTR contains the chopper domain (residues 277–308), linker region (309–338) and a death domain (DD) (residues 339–417)^10,11^. P75NTR forms covalent dimers via the transmembrane Cys257 ex vivo and in vivo and these dimers represent the functional receptor^12,13^. How these covalent dimers are activated by the p75 ligands is a matter of debate. The current models are based on the snail-tong mechanism^13^. It assumes that in the covalent p75 dimers, the DDs interact with each other, forming the homodimers and in response to NT binding there is a conformational change leading to dissociation of the DD homodimer allowing the interaction of the DD with intracellular adapter proteins^13,14^. This model is supported by the X-ray crystallography structures of p75DD dimers^15^, by the NMR titration experiments showing p75DD homodimerization^16^ and by the recently solved structure of p75DD dimers by NMR^14^. In addition, using the homo-FRET spectroscopy the same authors estimated the K_d_ of dimerization (49 ± 15 μM) and concluded that p75NTR DD forms low-affinity, noncovalent homodimers. Furthermore, the existence of p75NTR trimers was showed by Western blot analysis^17^.

On the other hand, the first structural study of intracellular domain of rat P75NTR found no sign of DDs self‐association^18^. Moreover, we recently showed by NMR that DDs of rat p75NTR do not interact with each other in the context of the rat p75NTR with the deletion of ECD (rP75-ΔECD) inside the lipid-protein nanodiscs^19,20^.

This inconsistency forced us to consider the DDs of p75NTR more deeply. One could suggest four major hypotheses to explain the listed findings: (1) the interface of the DDs dimerization is available only for isolated DD, but not in context of p75-ΔECD or full-length receptor, (2) rat and human p75NTRs behave differently, (3) formation of disulfide-crosslinked dimer is necessary to induce the interaction between the DDs in p75-ΔECD, (4) interaction between the DDs in full-length receptor may be observed only upon ligand binding. To test all the options, we investigated the homodimerization of human and rat p75NTR death domains in various protein contexts and under almost native ambient conditions, including isolated DDs, the disulfide bond cross-linked dimer of p75NTR with deleted extracellular domains inside the lipid-protein nanodisc particles and liposomes and the constitutively active p75 T249C mutant described in^21^.

## Results

### Isolated rat p75NTR death domains do not homodimerize

As a first step, we tested the hypothesis (1) by investigating the rat p75NTR DD. We have already shown previously that rat DDs are monomeric^20^, however, the studied construct contained long unstructured fragment of p75NTR, which could hinder the intermolecular contacts. Thus, we synthesized the fragment 331–425 directly corresponding to the DD of wild-type rat p75NTR and cleaved the polyhistidine tag (rP75DD). The construct was identical to the human DD, which was previously shown to form dimers^14^. Since the interactions between the DDs were reported to occur predominantly in phosphate buffers^14^, the structure of rP75DD was investigated in 50 mM NaPi, pH 7.0 in the presence of a reducing agent. To determine the oligomeric state of the protein this time we applied a direct approach instead of the chemical shift tracking—measured the rotational and translational diffusion by NMR at various concentrations up to 1 mM, which is an order of magnitude higher than the presumable K_d_. The resulting data were as follows at 1 mM concentration: rotational correlation time (τ_c_) was equal to 6.03 ± 0.23 ns and self-diffusion coefficient at infinite dilution (D_0_) was equal to (154 ± 2) × 10^–12^ m^2^ s^−1^ (all hydrodynamic parameters of tested DD constructs are summarized in Tables 1 and Supplementary Table S1). According to the Stokes–Einstein relationship these values correspond to the hydrodynamic radii (r_h_) of 1.96 and 1.80 nm, respectively, which is a good agreement. These radii may be converted into the molecular mass of a globular protein using a well-established relationship^22^ to obtain the protein weight of 13.0–10.1 kDa. Taking into account that the weight of the synthesized rP75DD is 10.5 kDa, we can conclude that the protein is entirely in the monomeric state. This agrees well with the results of hydrodynamic modeling. According to the HYDRONMR software^23^, the death domain of rat p75NTR (PDB ID: 1NGR^18^) should be characterized by the τ_c_ value of 5.8 ns and D_0_ of 146 × 10^–12^ m^2^ s^−1^, which excellently agrees with the experimentally observed number.

**Table 1:** Hydrodynamic parameters of various p75 DD constructs.

Construct/sample	τ_c_, ns	D_0_ × 10^–12^ m^2^ s^−1^	Predicted Mw^a^	Mw
Rat DD (1 mM)	6.0 ± 0.2	154 ± 2	13.0/10.2	10.6
Rat DD C416S (0.5 mM)	6.1 ± 0.2	151 ± 1	13.3/10.9	10.6
Rat DD C416S cross-linked	10.3 ± 0.3	123 ± 1	22.5/19.9	21.2
Human DD, 1 mM	6.1 ± 0.2	151 ± 2	13.3/10.9	10.5
Human DD, 1st state, 1 mM	6.4 ± 0.4	n.a.^b^	14.1/–	10.5
Human DD, 2nd state, 1 mM	6.0 ± 0.3	n.a.^b^	13.0/–	10.5

^a^Molecular weight was predicted based on the hydrodynamic radius of the equivalent sphere according to the work^22^. The first number is estimated based on rotational diffusion/second number is obtained from the D_0_ values.

^b^Translational diffusion cannot be measured for the two states separately.

To prove that the proposed approach is valid, we designed a positive control experiment and measured the diffusion of a disulfide-linked rP75DD dimer. We mutated the C416 residue of rP75DD to serine and the remaining single cysteine at position 379 was utilized for the cross-linking, activated by 2,2′-dithiobis(5-nitropyridine)^24^. The mutation did not change the overall appearance of the monomer HSQC NMR spectra, indicating that the fold of DD is retained. The measured diffusion parameters showed a dramatic change of τ_c_ and D_0_, which were corresponding to the protein with a molecular weight of more than 20 kDa (Table 1, Supplementary Table S1). These data are in excellent agreement with hydrodynamic calculations made for the PDB ID 4F44 (C379 disulfide-linked dimer of rat p75^15^) (Supplementary Table S1). Therefore, rotational and translational diffusion are reliable parameters that could be utilized to determine the oligomeric state of p75 death domains.

### Isolated human p75NTR death domains do not homodimerize either

**Figure 1:**
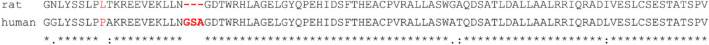
Comparison of rat and human death domain amino acid sequences. An * (asterisk) indicates fully conserved residue. A : (colon) indicates conservation between groups of strongly similar properties. A . (period) indicates conservation between groups of weakly similar properties. The non-conservative changes are in red. The additional amino acids in the human sequence are in bold.

The observed inability of rat DDs to self-associate at millimolar concentration should imply that rat and human proteins demonstrate the distinct behavior. This is rather unlikely because there is a very high homology between the rat and human p75NTR DDs sequences. There are only nine amino acid changes, and five of them are synonymous (Fig. 1).

Therefore, we decided to re-investigate the human p75NTR and synthesized the hP75DD construct (residues 330–427) identical to the one used by Lin et al.^14^. They observed two sets of signals, interpreted as the monomer (in HEPES buffer) and dimer (in phosphate buffer) states. In agreement, we also observed two sets of signals simultaneously in the phosphate buffer, the pattern of signals was visually identical to reported by Lin et al. (direct comparison is not possible since only one set (dimeric) is deposited to the PDB) (Supplementary Fig S1A). However, neither the change of buffer to HEPES nor the threefold dilution down to 300 μM of protein altered the population of states, which excludes the reversible dimerization. Moreover, we observed that the abundance of one state increased over time, while the population of the other decreased, which suggests an irreversible process. Finally, our measurements of diffusion coefficients in phosphate buffer clearly showed that both states correspond to the monomeric form of hP75DD (Table 1, Supplementary Table S1). These observations were further confirmed by the dynamic light scattering, which showed the r_h_ of hP75DD equal to 1.8 ± 0.19 nm (corresponds to Mw of 10.1 kDa) in samples with protein concentration up to 300 μM (Supplementary Fig S1C).

Thus, we show that (1) even at extremely high concentrations (1 mM), DDs of human p75NTR are monomeric, (2) the protein is transferred into the second state over time and this is an irreversible process, (3) the second state also corresponds to the monomeric form of DD. There is only one explanation of these facts—the irreversible modification of the protein monomer takes place. In turn, this resolves the apparent inconsistency between the behavior of human and rat receptors.

### Deamidation of p75NTR

**Figure 2:**
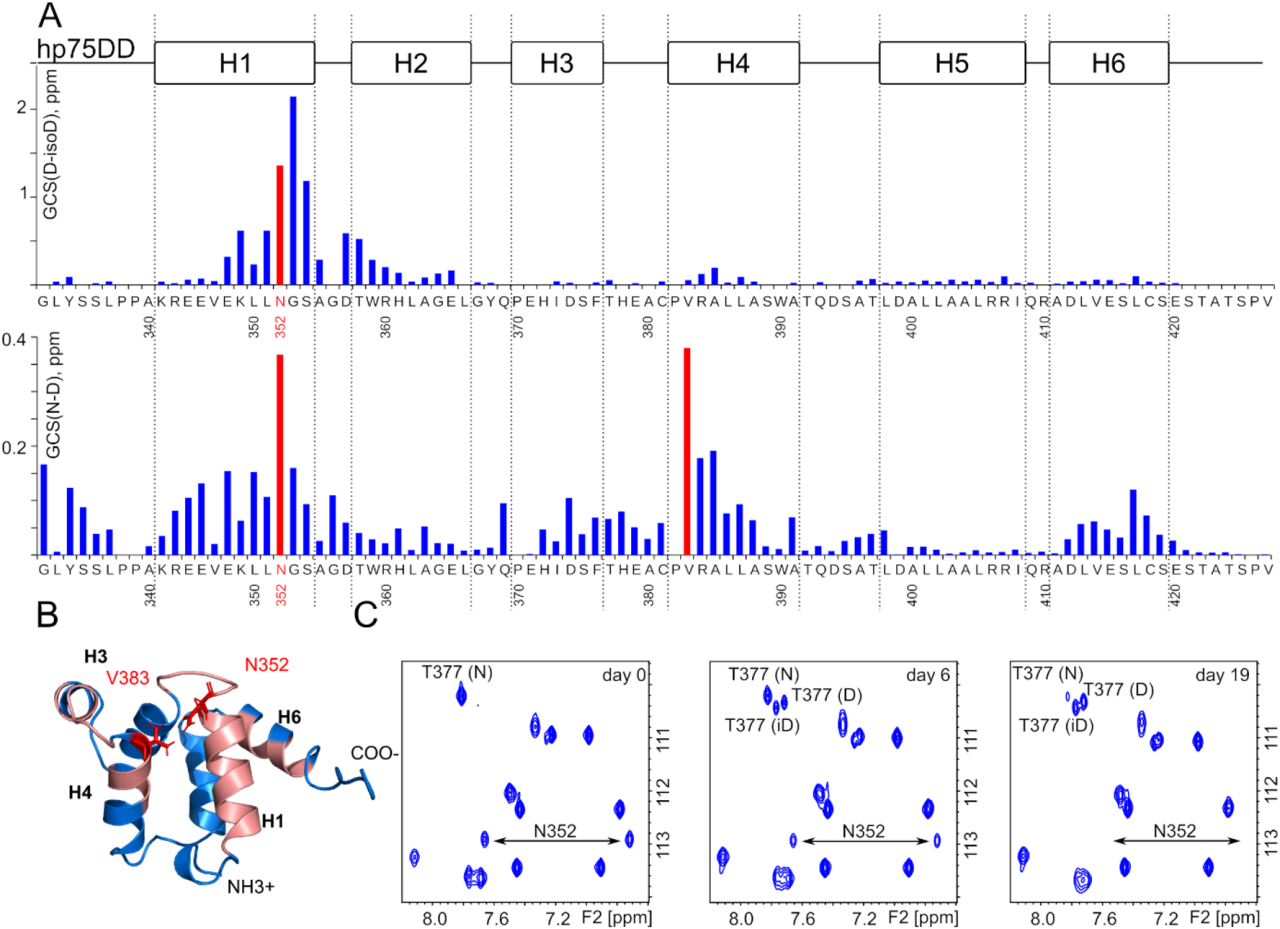
Analysis of the death domain states by NMR. (**A**) Generalized chemical shift changes of cross-peaks in ^1^H,^15^N-HSQC spectra between the D352 and isoD352 (on top) and between N352 and D352 (at bottom) states of hP75DD. (**B**) The structure of the death domain (PDB:2N97) is colored with respect to the chemical shift changes upon deamidation. Red are the residues with GCS changes greater than 0.1 ppm. (**C**) A set of three fragments of ^1^H,^15^N-HSQC spectra of hP75DD in 50 mM phosphate buffer acquired at immediately, on the 6th day and 19th day after the sample preparation. Positions of Asn352 sidechain NH_2_-group are shown, as well as of three well-resolved signals, corresponding to the different states of Thr377. The figure was prepared using the program Inkscape 0.92 (https://inkscape.org/).

**Figure 3:**
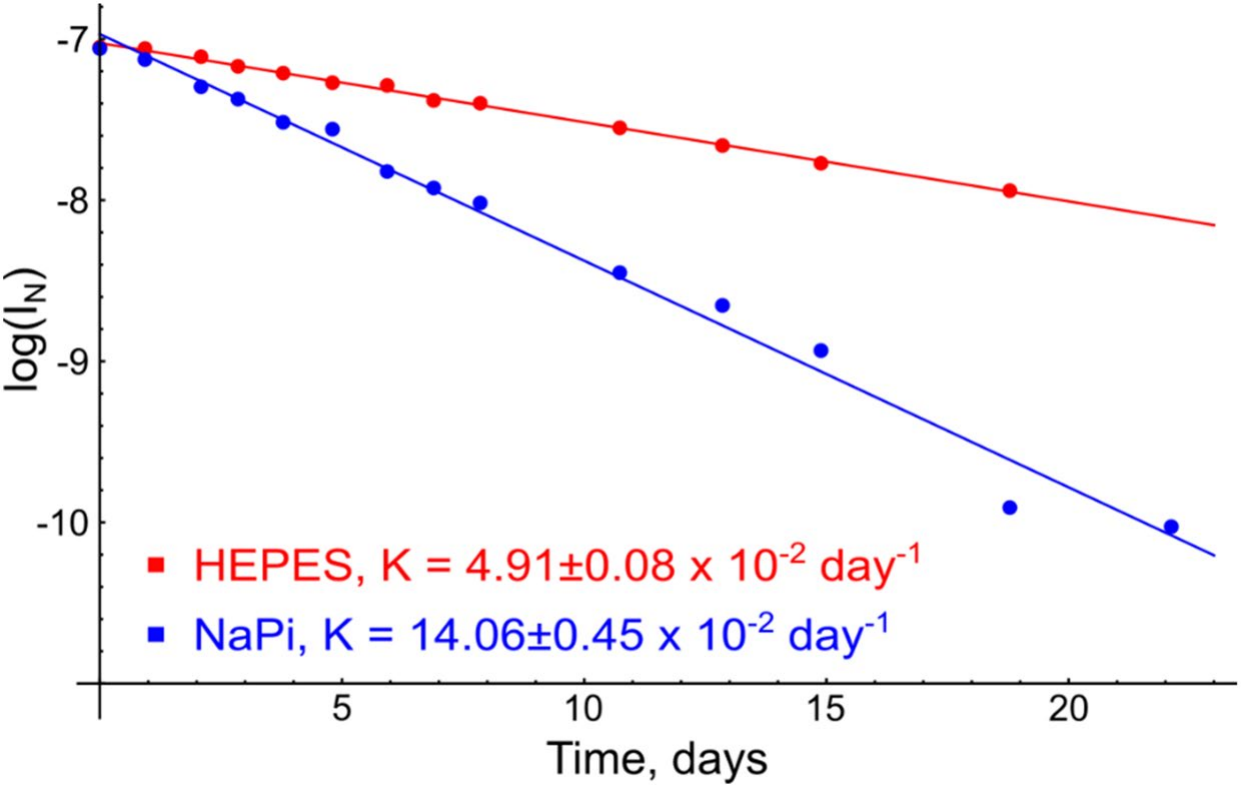
The kinetics of hP75DD deamidation in phosphate and HEPES buffers. The intensity of signal, corresponding to the N352 form of hP75DD is plotted as a function of time in a logarithmic scale. Two experiments are shown: in 20 mM HEPES pH 7.0 (in red) and 50 mM NaPi, pH 7.0 (in blue). The Figure was prepared using the program Inkscape 0.92 (https://inkscape.org/).

Since the uncatalyzed modification of p75NTR DD is observed in vitro, we considered it necessary to understand the nature of the phenomenon. The modified state occurs exclusively in phosphate buffer, therefore the obvious idea is spontaneous phosphorylation. With this in mind, we made an LC–MS analysis of the sample with two states of DD present. However, we observed a single peak on the MS, with no shift, characteristic for the phosphorylation. It means that the modification is accompanied by a small change of molecular weight, which could be lost in the broad isotope distribution of a ^15^N-labeled sample. Thus, we prepared two samples of hP75DD: (1) in HEPES buffer and (2) in phosphate buffer, measured kinetics of the modification and assigned the NMR chemical shifts, corresponding to the initial and final modified states of the protein. First, our assignment revealed that two states of the protein are formed at the end of the modification process, with highly different chemical shifts. Second, chemical shift difference is maximal for N352 and G353, reaching an enormous amount of 2.0 ppm for one of the states (Fig. 2). Third, the modification follows the first-order kinetics, the rate constant in phosphate buffer (14.06 ± 0.45 × 10^–2^ days^−1^, t_1/2_ ~ 5 days) being almost 3 times higher than in HEPES (4.91 ± 0.08 × 10^–2^ days^−1^, t_1/2_ ~ 14 days) (Fig. 3). Thinking of the process that can explain all our findings, we came to the idea of spontaneous deamidation of N352. The deamidation is fastest for the peptide sequence NG, is a first-order reaction, which is known to be catalyzed by phosphate ions and should result in the two forms of the protein, containing aspartic and isoaspartic acid in the corresponding position^25^. Finally, the molecular mass change upon deamidation is only 1 Da. Indeed, we found that the sidechain NH2 group of N352 disappears in the course of modification (Fig. 2). According to NOESY spectra, we assigned the state with isoaspartate in position 352, for this state the strongest peak was observed between the NH of G353 and C_β_H protons of now isoD352 (Supplementary Fig S2). To summarize, we have shown here that N352 of human DD undergoes a deamidation, accelerated in phosphate buffer with the domain remaining monomeric even after the modification.

### Activity and structure of triple Cys mutant of rat p75NTR

Although the data obtained here confirm that separate DDs do not interact with each other we decided to investigate the dimerization of rat p75NTR DDs in the most native protein context of disulfide cross-linked dimer. In our previous study, we showed that DDs of the rat p75NTR with deleted ECD (rP75-ΔECD) do not interact in the membrane environment in the presence of a reducing agent. The latter was added to avoid the oligomerization of the protein because three cysteines in the ICD would form multiple non-native disulfide bridges under oxidizing conditions. To exclude this, we engineered the triple cysteine mutant of p75NTR, rP75-3CX, with all the cysteines in the ICD (279, 379 and 416) being substituted by serines and only the Cys257 in TMD being retained. This allows assembling the dimer cross-linked via the transmembrane Cys257, avoiding the high-order oligomerization. However, to validate the approach, we first need to show that these mutations do not affect the activity of p75NTR and the structure of its DD.

**Figure 4:**
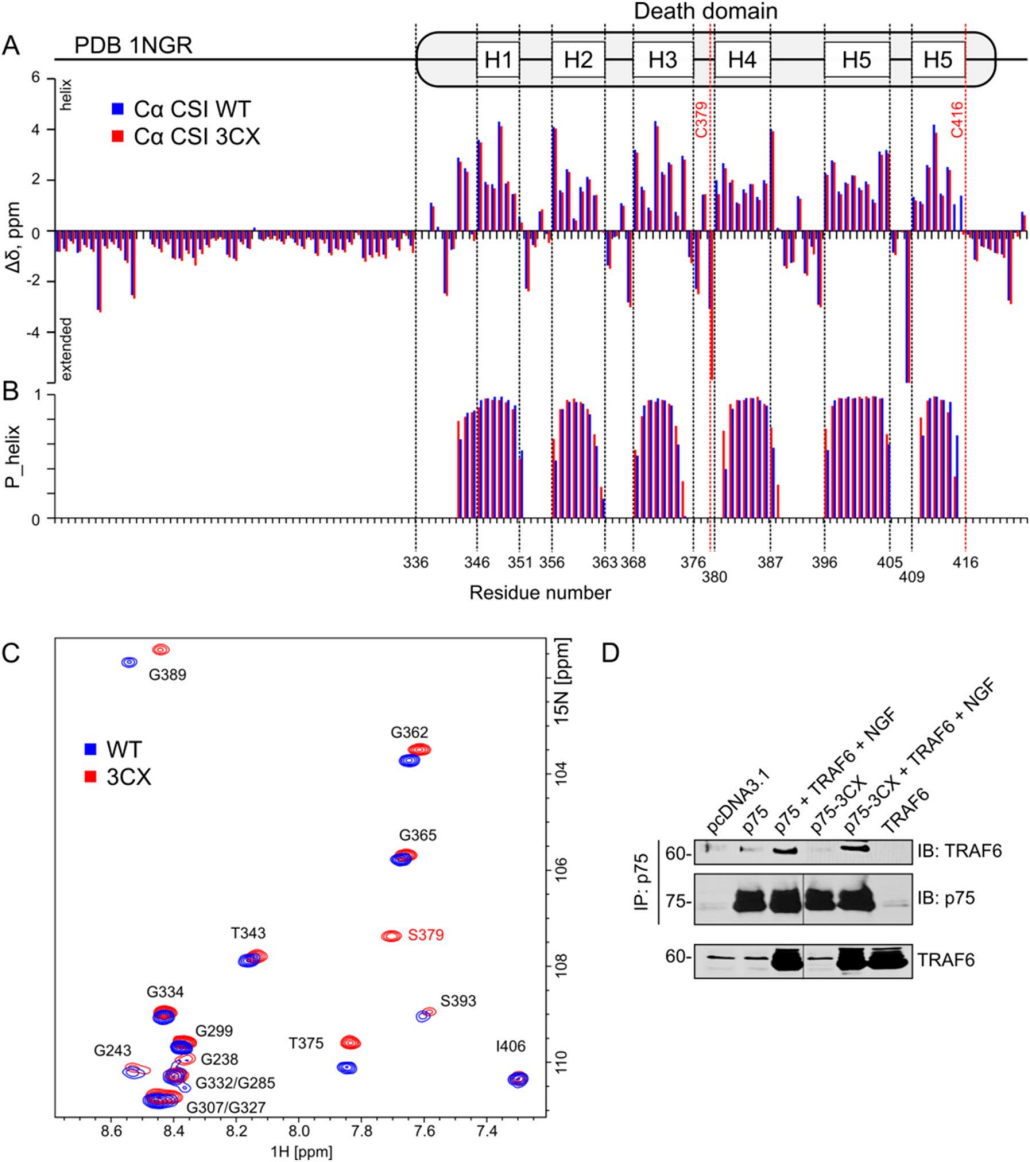
Comparison of structure and activity of wt and 3CX mutant forms of rP75NTR. (**A**) Secondary chemical shifts of C_α_ nuclei of rP75-ΔECD (blue bars) and rP75-ΔECD-3CX (red bars). The secondary structure of the death domain, according to PDB ID 1NGR is shown on top. (**B**) The propensity of ɑ-helical conformation, according to the TALOS-N prediction, based on NMR chemical shifts of rP75-ΔECD (blue bars) and rP75-ΔECD-3CX (red bars). (**C**) Overlay of ^1^H,^15^N-TROSY-HSQC spectra (glycine regions) of rP75-ΔECD (blue) and rP75-ΔECD-3CX (red). The assignment is provided in black and red (for the mutated S379 residue). (**D**) Binding of TRAF6 to wild type and mutant rP75-3CX in transfected HEK293 cells. Full-length blots are presented in Supplementary Fig S8. The figure was prepared using the program Inkscape 0.92 (https://inkscape.org/).

We used co-immunoprecipitation of TRAF6 upon NGF stimulation as a functional assay of rP75-3CX mutant^26^. As shown in Fig. 4D, rP75-3CX is able to co-immunoprecipitate with TRAF6 upon NGF stimulation to the same extent as the wild type variant of rat p75NTR. This result suggests that the rP75-3CX behaves like the p75-wt and the mutations do not impair the protein ability to interact with the TRAF6 adapter protein. Moreover, we analyzed the RhoGDI (PDB 2N83), RIP2 (PDB 2N80)^14^ binding interfaces and residues found important for the cell death signaling by mutagenesis^27^ (Supplementary Fig S10). Both Cys are not involved in the cell death signaling, but Cys418 (416 in rats) is on the RIP2 binding interface but does not form any hydrogen bonds. Thus, a synonymous C/S mutation should not impair the interaction.

Finally, we had to check the rP75-3CX structural properties. We mutated the rat rP75-ΔECD construct to create rP75-ΔECD-3CX for bacterial expression and placed it into the lipid-protein nanodiscs, following the previously published protocol and studied its structure and dynamics by NMR^20^. We managed to assign the backbone signals of 90% of ICD, except for the first 9 (K274-N282) juxtamembrane residues, which are likely to be immobilized on the membrane surface. According to the NMR chemical shift data no significant changes in the secondary structure compared with wild type rP75-ΔECD were detected (Fig. 4B). Some discrepancies in the secondary chemical shifts are observed in close proximity to the mutated Cys residues (Fig. 4A,C). However, they are not reflected in the consensus secondary structure prediction by the TALOS-N software, which takes into account not only the residue type but also the context in amino acid sequence^28^. According to the cross-correlated relaxation rates^29^, the DD of rP75-ΔECD-3CX demonstrates almost the same average rates of rotational diffusion (5.78 ± 0.16 ns) as the domain of wild-type protein (6.24 ± 0.2 ns). The NMR relaxation data confirmed that the chopper domain is flexible and disordered and the mobility of DD corresponds to a 12.9–13.5 kDa globular water-soluble protein^22^.

To summarize, we have shown that rP75-ΔECD-3CX mutant of the rat p75NTR has the same functionality as wild type ex vivo and reveals no significant changes in its ICD structure in vitro.

### Rat p75NTR death domains do not interact in the context of Cys257 SS-linked dimer

Since activity and structure of rP75-3CX are similar to the wild type receptor we can investigate the interaction between DDs in the C257 covalent dimer under non-reducing conditions. For this purpose, we synthesized the rP75-ΔECD-3CX construct, pre-dimerized it, placed into the LPNs (Supplementary Fig S3) and investigated by NMR spectroscopy.

**Figure 5:**
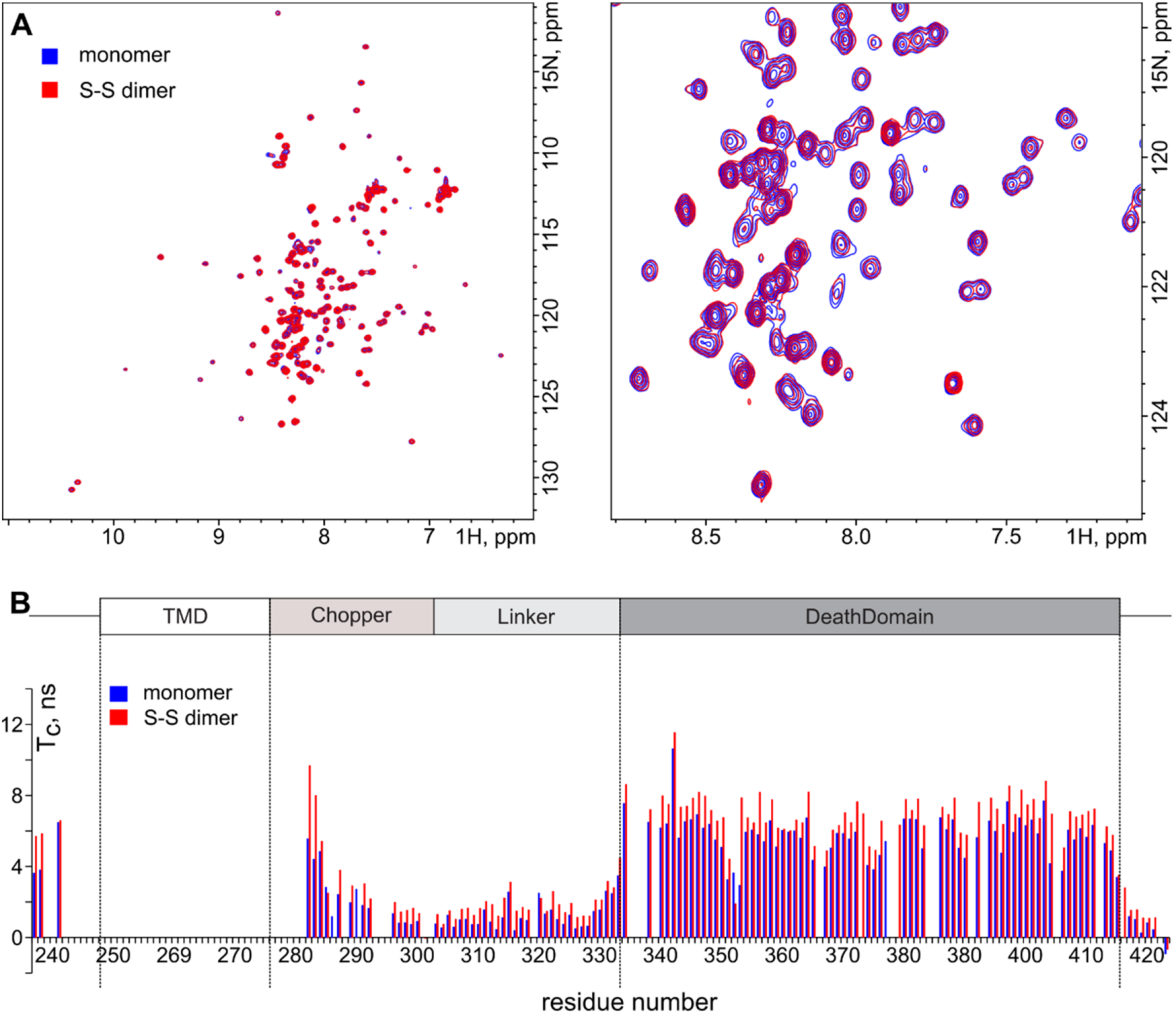
The effect of dimerization on the structure and behavior of the intracellular domain of rP75-ΔECD-3CX. (**A**) ^1^H,^15^N-HSQC spectra of rP75-ΔECD-3CX monomer (shown in blue) and disulfide-crosslinked dimer (shown in red). The full spectrum and central region are shown at left and right, respectively. (**B**) NMR-derived rotational diffusion correlation time of N–H bonds is plotted versus the residue number of rP75-ΔECD-3CX monomer (blue bars) and dimer (red bars). The figure was prepared using the program Inkscape 0.92 (https://inkscape.org/).

First of all, ^1^H,^15^N-HSQC spectra revealed no difference between the dimer and monomer of rP75-ΔECD-3CX, except for the line broadening of several membrane-adjacent residues (Fig. 5A). This implies that within the disulfide-crosslinked dimer we observe the monomeric state of DD. Moreover, the intensities of the DD signals in NMR spectra did not change upon C257 dimerization. This excludes the presence of DD dimer, invisible in NMR spectra due to the extremely low mobility. To further assess the effect of covalent dimerization on the behavior of p75NTR ICDs, we measured the τ_c_ of amide groups (Fig. 5B). This experiment revealed that the mobility of the DD is decreased by 1.05 ± 0.06 ns (15%) due to the cross-linking of rP75-ΔECD (Fig. 5B). The change is too subtle to be caused by the direct self-association of the domains. On the other hand, while the linker region is still highly flexible, the motions of this part of rP75-ΔECD become slightly slower in the disulfide dimer. We assume, that the small deceleration effect of DDs is due to the restricted conformational space of the linker. Indeed, significant effects of cross-linking on the protein mobility are observed only at the closest proximity to the membrane of LPNs (Fig. 5B).

Thus, we can conclude that no specific interaction between the DDs of p75NTR is observed in the context of a disulfide-crosslinked dimer of rP75-ΔECD construct. The linker region remains flexible, no structural changes are detected. The dimerization of the construct results only in a subtle decrease in the mobility of the DD and linker regions. The state of TM domain may cause an effect on only the first 12 residues of the membrane-adjacent region of ICD.

### Rat p75NTR death domains do not interact with bilayer membranes

**Figure 6:**
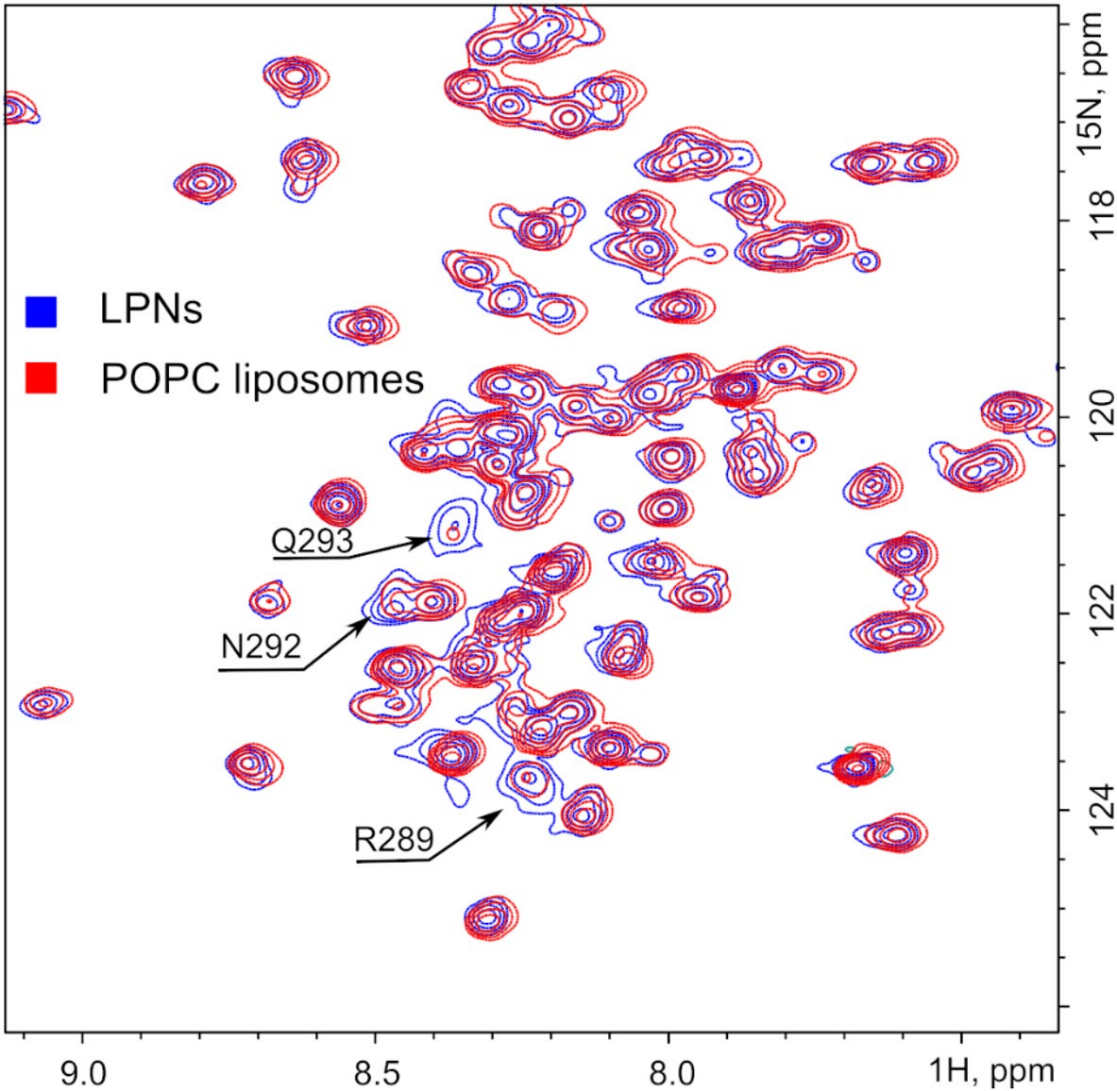
NMR analysis of rP75-ΔECD-3CX incorporated in nanodiscs and POPC liposomes. Fragment of 2D-^1^H,^15^N-TROSY-HSQC spectrum of dimeric rP75-ΔECD-3CX in MSP1D1 LPNs (shown in blue) and POPC liposomes (red). Both samples were prepared at lipid-to-protein ratio (LPR) 150. Assignments of amide group signals with the visible line broadening are indicated. The figure was prepared using the program Inkscape 0.92 (https://inkscape.org/).

While in LPNs and bicelles the linker region is flexible and DDs of p75NTR do not interact, the situation might be different in real bilayer membranes, where the intrinsically disordered region can become structured due to the protein–membrane interactions. To test this possibility, we prepared the rP75-ΔECD-3CX SS-linked dimer sample incorporated into the DMPC and POPC liposomes as one of the most native membrane-like environment. While the size of liposomes is too high for NMR structural applications the disorder and flexibility of the chopper domain as well as high mobility of DD allowed obtaining the structural information about the ICD. First of all, the mere fact that we are able to observe the NMR spectra of DDs implies that motions of the domains are uncoupled from the motions of TMD helix. Next, NMR chemical shifts are identical for the DD and part of the linker domain of rP75-ΔECD-3CX in LPNs and both types of liposomes. Signals of several juxtamembrane residues become broadened in liposomes, indicating that the influence of membrane is propagated farther than we had originally assumed—up to 20 amino acids apart from the membrane surface (Fig. 6). NMR relaxation data did not reveal any substantial difference between the LPN and POPC samples. The DD motions were on average 0.11 ± 0.12 ns faster in liposomes, which is insignificant and negligible. The linker region remained highly flexible (Supplementary Fig S4).

Thus, we can conclude that our LPN system provides an adequate environment for rP75-ΔECD. The presence of an extended patch of bilayer membrane does not cause any structuring of the linker region or significant changes in the p75NTR ICD behavior, including the propensity for homodimerization. In summary, the DDs interact neither with each other nor with the bilayer membrane formed by POPC or DMPC lipids.

### Receptor-activating p75-T249C mutation does not affect the behavior of p75 intracellular domain

We did not observe the interaction between the rat p75NTR DDs within the SS-linked dimer of rP75-ΔECD inside LPNs. However, there is still a possibility that domains could self-associate upon the receptor activation. Earlier Vilar et al. reported several mutations in the extracellular juxtamembrane region that caused the ligand-independent signaling by p75NTR^21^. We decided to investigate one of these mutations (T249C) and posed the question: how can it affect the ICD behavior?

We produced the rP75-ΔECD-3CX-249C construct in the dimeric state and studied it similarly to rP75-ΔECD-3CX in DMPC LPNs. No changes were observed in NMR spectra of the intracellular domains, compared to the rP75-ΔECD-3CX dimer sample. To assess the effect of C249 cross-linking, we added a reducing agent, TCEP, to the solution. After this most of the protein passed to the monomeric state, according to the SDS-page. In contrast, only very subtle changes were found in NMR spectra, moreover, all of them taking place for the N-terminal residues: G243, R241, and V240 (residues 244–282, that include the TMD, are not observed due to the low mobility of LPN). Thus, the C249–C249 SS-bond, which mimics the ligand-binding by p75NTR, does not affect the properties of the intracellular part of the receptor (Supplementary Fig S5).

### The T249C mutation increases the rate of the receptor dimerization/oligomerization

**Figure 7:**
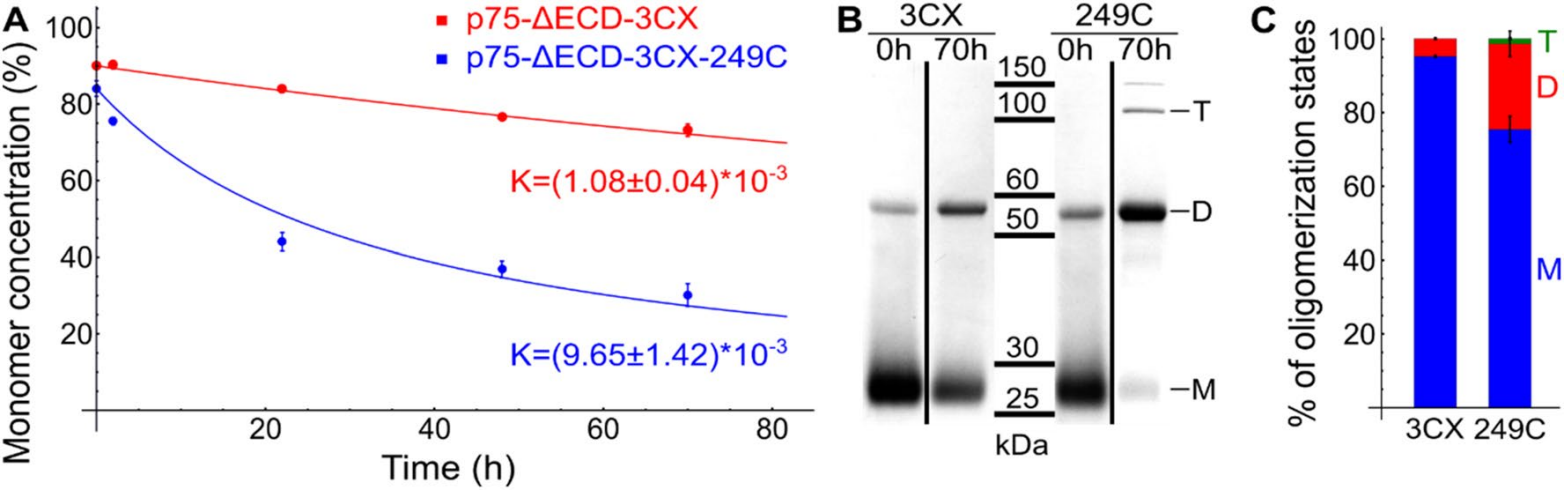
Comparison of rP75-ΔECD-3CX and rP75-ΔECD-3CX-249C oligomerization rates. (**A**) The kinetics of rP75-ΔECD-3CX and rP75-ΔECD-3CX-249C dimerization based on SDS-page analysis (n = 3). k—the reaction rate constant calculated for irreversible second-order reaction. (**B**) The SDS-page analysis of purified proteins incorporated into DMPC:CHAPS (q = 1, LPR = 1,000:1) bicelles at the initial state and after 70 h of incubation without reducing agents (after dialysis). Full-length gels are presented in Supplementary Fig S9. (**C**) Comparison of rP75-ΔECD-3CX and rP75-ΔECD-3CX-249C oligomerization in the membrane of *E. coli* cells. Both strains were grown under the same conditions and the equivalent amount of cells (normalized to OD600) were harvested through 72 h after induction. After cell lysis, the membrane fraction was separated, solubilized in SDS containing buffer and analyzed by SDS-PAGE. *3CX* rP75-ΔECD-3CX, *249C* rP75-ΔECD-3CX-249C, *M* monomer, *D* dimer, *T* tetramer. The figure was prepared using the program Inkscape 0.92 (https://inkscape.org/).

While the T249C mutation had no effect on rP75-ΔECD structure and dynamics, we noticed that (1) the dimer formation was faster than in wild type and (2) rP75-ΔECD-3CX-249C formed several high-order oligomer forms. To describe this phenomenon in detail and estimate the kinetics of disulfide bond formation we produced two constructs, rP75-ΔECD-3CX-249C and rP75-ΔECD-3CX, and placed them into the DMPC/CHAPS q = 1 bicelles. This environment was shown to maintain the native fold of the p75NTR DD and provide the conditions close to the LPNs^19^. To estimate the amount of monomer and dimer/oligomer forms we utilized the non-reducing SDS-page analysis. First of all, we observed a significant increase in the dimerization rate for rP75-ΔECD-3CX-249C compared to rP75-ΔECD-3CX (Fig. 7). The obtained data were approximated by the simplest irreversible second-order kinetics to measure the dimerization rate constants, which appeared equal: (1.08 ± 0.04) × 10^–3^ and (9.65 ± 1.42) × 10^–3^ (mole_lipid_/mole_protein_)s^−1^, for rP75-ΔECD-3CX and rP75-ΔECD-3CX-249C, respectively (Fig. 7A).

Second, the T249C mutant formed several abundant high-order oligomers (Fig. 7B). The major form is still dimer, however, the presence of tetramers, hexamers etc. indicates that the extracellular juxtamembrane region is flexible and the SH-group of Cys249 can form a disulfide bridge with the third p75NTR molecule.

Moreover, we cultured the bacterial strains expressing rP75-ΔECD-3CX and rP75-ΔECD-3CX-249C under identical conditions and analyzed the membrane fractions of *E. coli* by SDS-PAGE. Similar behavior was observed: the rP75-ΔECD-3CX-249C formed more dimers, compared to the rP75-ΔECD-3CX (Fig. 7C, Supplementary Fig S6).

While the dimerization is clearly observed for the T249C mutant receptor and the wild type, we need to note that this process is relatively slow. Indeed, in bicelles with an LPR value of 1,000:1 we had ~ 50% of rP75-ΔECD-3CX-249C oligomers and less than 20% of a wild type after 20 h of incubation. In *E. coli* experiments the LPR value for the membrane fraction could be estimated as ~ 200:1, but the main form of the receptor was monomer even after 3 days of cell cultivation. It is obvious that at high LPR the rate of dimerization should be slower and vice versa. Indeed, we repeated our experiment in bicelles at 5 times lower LPR value (200:1) and found that the dimerization rate becomes significantly higher for both the mutant receptor and wild type (Supplementary Fig S6B). Thus, since the protein concentrations under native conditions in neurons are much lower, the dimerization rate should be many times slower than in our experiments and the self-formation of a small amount of dimer can take weeks.

Thus we have shown that the disulfide formation rate for the T249C mutant is significantly faster than for the wild type and that this mutant can form dimers and oligomers of higher order in vitro and ex vivo, but the absolute values of self-association rates at low protein concentration are very low.

## Discussion

**Figure 8:**
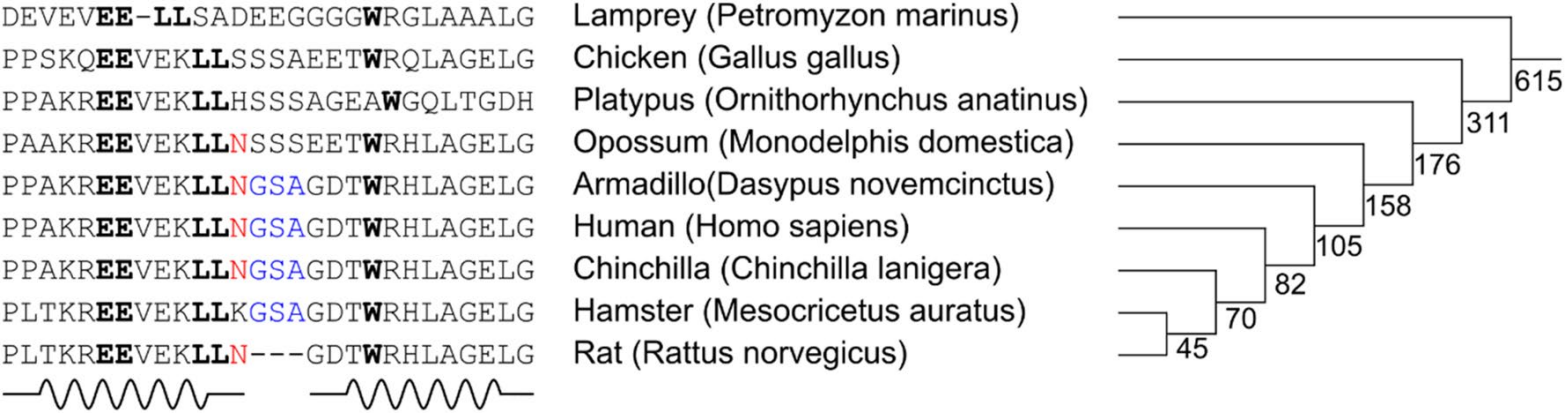
Sequence alignment of death domain fragments from different organisms. The highly conserved residues are in bold. The N352 is shown by red, GSA sequence by blue. The secondary structure of the rat p75NTR is shown under amino acid sequences. The phylogenetic tree (right) shows the evolutionary history of the DD fragment of the p75NTR gene, the numbers indicate the split time of species (in a million years, according to the Ensembl data: https://www.ensembl.org/Multi/GeneTree/Image?gt=ENSGT00730000110974).

Here we found that N352 of human p75NTR is deamidated readily and some components of the environment like phosphate ions can speed up this process by several times. On the other hand, for the case of rat p75, no deamidation was observed. A comparison of the amino acid sequences reveals a 3-residue (GSA) insert in the human protein after the N352 (Fig. 1). It is well known that NG sequence is at most prone to deamidation^25^. Interestingly, while rat DD does not include the GSA sequence, the next amino acid after N353 is still G too, but it is not enough for the deamidation according to our data. N353 in the rat domain is located at the beginning of a linker between the helices H1 and H2 (Fig. 8). The linker consists of only four amino acids, however, it is rather flexible, according to our relaxation measurements^20^, heteronuclear NOE values for N353 and G354 are dropped substantially, compared to the neighboring residues (Supplementary Fig S7). On the other hand, in human DD helix H1 is two residues longer and includes both N352 and G353, which are now rigid. Moreover, according to the NOE data, the preferred sidechain χ_1_ conformation of N352 is 180°, which directs the amide group of asparagine towards the carbonyl of the residue and amide proton of G354. Since the initial step of deamidation is the cyclization of Asn sidechain to the amide group of the following residue, we can conclude that the GSA insert in human p75 stabilizes the conformation of N^353^G^354^ fragment in the state, favorable for the start of deamidation.

N352 modification is difficult to link with the protein function because there is no biological data like functional studies or mutagenesis focused on this protein region, but it can play some role. From the evolutionary point of view, N352 first appeared about 175 million years ago with the advent of mammals and after 20 million years the GSA sequence was “added” after it (Fig. 8). Since that time this sequence did not change in most organisms, excluding only two rodents branches (Fig. 8). There are several studies showing the importance of deamidation. It has been proposed to represent a “molecular timer” determining the lifetime of proteins^30–33^. The deamidation affects the structural integrity and biological activity of proteins and it was associated with some pathologies including neurodegenerative disorders^34–38^. Moreover, the process of Asn deamidation has a reverse reaction catalyzed by isoaspartate methyltransferase also known as PIMT. It turns isoAsp to Asp residue and may help the protein to regain its function^39^.

Taking into account all aforesaid we propose that the evolutionary selection of this modification might be not accidental and could be involved in some biological function: the modification may act as the trigger of protein turnover or affect the interactions with adapter proteins. Additionally, now most researchers study the p75NTR function using various models—rat, murine and human. However, as we have shown here, human and rat proteins may behave differently due to the observed deamidation, therefore some care has to be taken in establishing the equivalence of signaling events for these systems.

But what is the basis of p75NTR functioning in the light of new data? Earlier we showed that death domains (DDs) of the rat p75 do not interact in p75-ΔECD constructs placed into the same LPN^20^. Here we extend our data and show that death domains do not interact in any context. Experiments with isolated rat and human DDs revealed that they do not homodimerize in solution up to 1 mM concentration. Studies in more native context—in LPNs and liposomes showed that membrane environment and covalent dimerization by C257 have no detectable effect on the receptor structure and there is still no interactions between the DDs. Moreover, the receptor-activating mutation T249C^21^ does not affect the DDs in any way. We acknowledge that our data was obtained in vitro using the *E. coli* produced proteins, which may affect the folding and functions of the domain and distort the results. However, taking into account that there are no known sites for posttranslational modifications within the DD and that the DD structure is very similar to many previously studied (there are 46 death domain structures in PDB), we believe that the data obtained here reflect the native state of the death domain.

All of these observations allow us to claim that DDs are not capable of homodimerization. The keystone of the “snail-tong” mechanism of p75 activation is that ligand binding causes an interaction between the extracellular domains and release of the intracellular death domains from the homodimer complex. This implies two assumptions: DDs of p75NTR can homodimerize, and conformation of DDs can be sensitive to the ligand binding. Both assumptions are not supported by our data. Therefore, we conclude that the snail-tong hypothesis should be revised. We need to suggest a mechanism that does not require the direct coupling between the states of TMDs and DDs, as well as the DD dimerization.

We need to note that most ex vivo investigations on non-neuronal cells show that the main form of p75NTR is monomeric under the overexpression conditions^13,40,41^. Moreover, here we show that while p75-ΔECD is prone to dimerization, the rate of this process is very slow even at relatively low LPR values (Fig. 7, Supplementary Fig S6). On the other hand, only C257 covalent dimers of the p75NTR were detected in neuronal cell lines at endogenous expression level^13^. According to the most basic laws of chemical kinetics, the increase in concentration should result in the increased abundance of the dimeric state, however, the opposite is observed. This fact can be explained in two ways: (1) there is a protease that degrades only the monomer form of p75NTR and (2) there is a “helper” protein that catalyzes the dimerization. Both hypotheses require an additional participant for the accumulation of a covalent dimer. Indeed, if the expression of p75NTR is low, like in native conditions, the rate of spontaneous dimerization would be very slow and mainly monomers would be detected. The protease that degrades only the monomeric form of p75NTR can also cause the dimer accumulation, but the possibility of this process depends on three parameters: the rate of monomer degradation by protease, the rate of receptor expression and the rate of dimer proteolysis. The absence of detectable amounts of monomers in neurons means that the rate of protease activity should be much higher than the rate of protein production. Taking into account a low rate of spontaneous dimerization we conclude that the rate of dimer proteolysis has to be extremely low compared to the one of a monomer. Moreover, p75NTR is a well-known substrate for γ-secretase. However, the cleavage does not regulate the receptor dimerization, therefore the propensity of p75 to form dimers mainly at low expression levels cannot be explained by the protease activity^41^.

**Figure 9:**
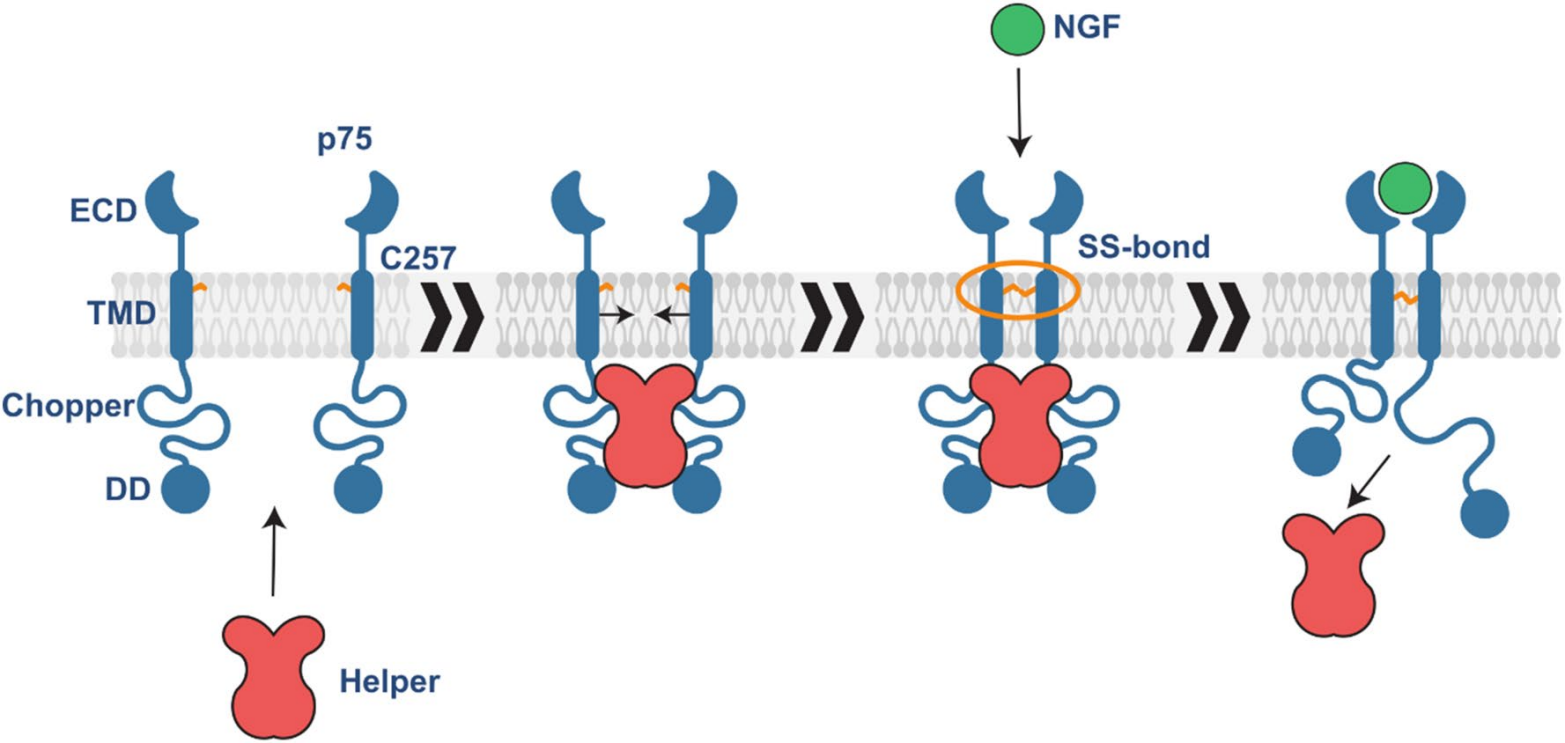
The mechanism of p75NTR dimerization and activation upon the ligand binding. Schematic representation of p75NTR covalent dimer formation in membrane by “helper” protein and receptor activation in response to neurotrophin binding. The figure was prepared using the program Inkscape 0.92 (https://inkscape.org/).

Thus, we state that the most probable explanation of the covalent dimer accumulation on the membrane surface is the second hypothesis, implying that the “helper” protein interacts with P75NTR molecules and keeps them in close proximity to each other. This leads to disulfide cross-linking and the presence of a complex formed by the p75NTR dimer and “helper” (Fig. 9).

Upon the p75NTR overexpression, the amount of “helper” is not enough to bind all the p75NTR molecules and the monomers of the receptor are detected. Moreover, the “helper” can play an additional role in the p75NTR signaling—to prevent the p75NTR from interactions with adapter proteins. Upon ligand binding, conformational changes occur, the “helper” is released from the complex and the ability of P75NTR to interact with the adapter proteins is restored. Homo-FRET experiments have shown that the ICDs in the p75NTR dimer are in close proximity to each other under basal conditions but upon NGF binding the FRET signal decreases^13^. This observation can be explained by the interaction with “helper”, which can bring ICDs closer together, but when “helper” is released, the ICDs of p75NTR become mobile as we observe in our NMR experiments.

One can now pose a reasonable question: What is the “helper” protein? It can be a membrane protein that interacts with ECD of p75NTR and is thus sensitive to NGF binding. Caveolin, Sortilin, SORCS2, NgR, Lingo-1, and TrkA are known membrane proteins interacting with p75NTR^42–48^. However, interaction with ECD would not explain the absence of receptor activity without the NGF at normal expression levels. On the other hand, here we investigated the structure of p75-ΔECD and p75-ΔECD-T249C covalent dimers and found that there are no interactions and structural changes in the ICD domain in response to the p75NTR dimerization or activating mutation. However, the TM and several first juxtamembrane residues are not detected by NMR due to the signal broadening. These parts of the receptor could be sensitive to the ligand-binding along with the ECD. Thus, the “helper” could interact with TM or juxtamembrane part of the p75NTR to react on the ligand binding. This implies that it is likely a membrane-associated or a soluble protein that can bind the juxtamembrane domain (JMD) of p75NTR (Fig. 9). To date there are several proteins that interact with JMD^44,49–53^, but there is insufficient information to define clearly the protein that could play the “helper” role and the additional studies are required.

## Conclusions

In the present work, we studied the interactions between the intracellular domains of p75 neurotrophin receptor with NMR and biochemical approaches. First of all, we showed that neither rat nor human p75NTR DDs homodimerize in solution. Second, we investigated the influence of covalent rP75-ΔECD dimerization on the DD dimerization and showed that there is no interaction between closely positioned DDs. Third, we produced the T249C mutant of rP75-ΔECD and showed that this mutation does not affect the behavior of the intracellular domain. Thus we postulate that there is no interaction between DDs of p75NTR in any context and we believe that the snail tong model does not agree with the experiment data and should be revised. Finally, we propose another model of P75NTR functioning, suggesting that P75NTR dimerization occurs in the complex of two P75NTR molecules and “helper” protein, and ligand binding leads to the “helper” release, which restores the ability of P75NTR dimer to interact with adapter proteins.

## Materials and methods

### Gene construction

Plasmid to express rP75-ΔECD fragment (residues 245–425) of the rat p75 and purification protocol were previously described^20^. Mutations C279S, C379S and C416S were introduced in rP75-ΔECD fragment by PCR to produce triple cysteine mutant rP75-ΔECD-3CX.

Mutation T249C was introduced in rP75-ΔECD-3CX gene by PCR to produce rP75-ΔECD-3CX-249C construction.

Target nucleotide sequences encoding the human (hP75DD, UNIPROT: P08138) and rat (rP75DD, UNIPROT: P07174) death domains were amplified by PCR based on human wild type p75NTR (purchased from Twist Bioscience with optimized codons for *E. coli* expression) and rat p75-ΔECD genes, respectively. The C416S mutation was introduced to rP75DD gene by PCR to produce rP75DD-416S construct.

All genes were cloned into pGEMEX1-derived expression vector between BamHI and HindIII restriction sites for the hybrid expression of target fragments. Each recombinant protein contains additional N-terminal residues (MHHHHHHGSGSGLVPRGS). All constructs were verified by DNA sequencing.

### Genes expression and protein purification

All proteins were expressed in *E. coli* strain BL21(DE3) in M9 medium. To obtain the ^15^N-labeled or ^15^N-^13^C-labeled proteins, we used ^15^NH_4_Cl or ^15^NH_4_Cl and [U-^13^C]-glucose, respectively. The cells were grown overnight at 28 °C in a shaking incubator (New Brunswick Innova 44R) at 250 rpm. Protein expression was induced at OD600 ~ 0.6 by isopropyl β-d-1-thiogalactopyranoside (IPTG) (Table 2). Expression was carried out for several hours (Table 2) and cells were harvested by centrifugation at 7,000×*g* for 10 min at 4 °C and stored at − 20 °C for later use.

**Table 2:** The cultivation conditions of E. coli for expression of rP75-ΔECD-3CX, rP75-ΔECD-3CX-249C, rP75DD and hP75DD proteins.

	rP75-ΔECD-3CX	rP75-ΔECD-3CX-249C	rP75DD	hP75DD
IPTG, mM ^a^	Without IPTG	0.1	0.5	0.5
Time, h ^b^	24–30	24–30	30	4
Temp., °C ^c^	37	13	13	37

^a^Final concentration of inductor, IPTG.

^b^The time of cells growth after induction.

^c^The temperature of cells cultivation after induction.

The rP75-ΔECD-3CX and rP75-ΔECD-3CX-249C proteins were purified according to a previously described protocol^20^. Briefly, the target protein was solubilized in lauryl sarcosine buffer, purified by metal affinity and size-exclusion chromatography. To prepare the SS-linked dimers we modified our sample preparation protocol. In order to bring the monomers closer and to accelerate the disulfide bond formation the purified protein (rP75-ΔECD-3CX or rP75-ΔECD-3CX-249C) was concentrated up to 0.3–0.5 mM by ultrafiltration (10 kDa MWCO, Amicon Ultra) and dialyzed against an aqueous buffer without detergent (20 mM Tris, pH 8.0, 100 mM NaCl) until most of the protein passed to the dimeric state. The amount of disulfide-linked dimers was estimated by non-reducing SDS-page. To separate the dimer and monomer, the size-exclusion chromatography in lauroylsarcosine buffer was repeated. Fractions with predominantly the dimer form were pooled.

The rP75DD and rP75DDC416S proteins were purified using metal affinity, ion-exchange and size-exclusion chromatography. The cells were resuspended in lysis buffer [20 mM Tris, pH 8.0, 500 mM NaCl, 100 µM phenylmethylsulfonyl fluoride (PMSF) and 10 mM β-mercaptoethanol (bME)] and disrupted by sonication (Bandelin Sonopuls) on ice 10 times at 65% amplitude with 15 s/3 min on/off program. The lysate was clarified by centrifugation at 14,000×*g* for 30 min and 150,000×*g* for 1 h at 4 °C. The supernatant was filtered using Millipore filter unit with 0.2 µm pore size and loaded to Ni Sepharose HP resin (GE) column, which was pre-equilibrated with IMAC-buffer (20 mM Tris, pH 8.0, 250 mM NaCl, 10 mM bME) with 20 mM imidazole. The impurities were removed by washing the column successively with 10 column volumes (CV) of IMAC-buffer with 20 mM and 40 mM imidazole. The target protein was eluted with IMAC-buffer with 400 mM imidazole. The fractions with target protein were analyzed by SDS-PAGE, pooled and dialyzed at 4 °C against Q-buffer (10 mM Tris, pH 8.5, 10 mM bME) with 0.005% NaN3. The precipitate was removed by centrifugation at 14,000×*g* for 30 min. The supernatant was filtered and loaded to Q Sepharose resin (GE) column, which was pre-equilibrated with Q-buffer. The resin was washed with 10 CV of Q-buffer and elution was carried out in Q-buffer using a salt gradient from 0 to 1 M of NaCl per 10 CV. Fractions with target protein were analyzed by SDS-PAGE and pooled. Protein was concentrated by ultrafiltration (10 kDa MWCO, Amicon Ultra) up to 6–7 mg ml^−1^, centrifuged at 25,000×*g* for 30 min and supernatant loaded to a Superdex 75 pg (GE) Tricorn 10/600 gel filtration column equilibrated in NMR buffer (50 mM NaPi, pH 7.0, 10 mM DTT, 1 mM EDTA, 0.01% NaN3). Fractions containing rP75DD (C416S) were analyzed by SDS-PAGE, pooled and concentrated by ultrafiltration up to 12–14 mg ml^−1^. To remove N-terminal histidine tag the protein was treated with 30 U mg^−1^ of thrombin overnight at 18 °C and the gel filtration step was repeated. To cross-link the rP75DDC416S, 1 mg of protein was incubated with a tenfold excess of 2,2′-dithiobis(5-nitropyridine)^24^ for 3 h in 500 µl of NMR buffer with mild shaking at 37 °C. This resulted in the obtaining of 60% protein dimer and 40% of rP75DDC416S modified by S-(5-nitropyridine). The solution was then dialyzed overnight and supplied by 0.4 mg of rP75DDC416S. The solution became yellow and 80–90% of target protein passed to the cross-linked state. The amount of protein in a cross-linked state was controlled by solution NMR and non-reducing SDS-PAGE.

The hP75DD protein was purified using metal affinity and size-exclusion chromatography. The cells were harvested by centrifugation at 7,000×*g* 7 min at 4 °C, resuspended in lysis buffer (20 mM HEPES, pH 8.0, 500 mM NaCl, 100 µM PMSF and 10 mM bME) and disrupted by sonication on ice 10 times at 65% amplitude with 15 s/3 min on/off program. The lysate was clarified by centrifugation at 14,000×*g* for 30 min and 150,000×*g* for 1 h at 4 °C. The supernatant was filtered using Millipore filter unit with 0.2 μm pore size and loaded to Ni Sepharose HP resin (GE) column, which was pre-equilibrated with hIMAC-buffer (20 mM HEPES, pH 8.0, 250 mM NaCl, 10 mM bME) with 20 mM imidazole. The impurities were removed by washing the column successively with 10 CV of hIMAC-buffer with 40 mM imidazole. To remove N-terminal histidine tag the column was equilibrated with thrombin-buffer (20 mM HEPES, pH 8.0, 10 mM bME, 150 mM NaCl, 0.001% NaN3), thrombin (10 U mg^−1^) (Technologia-Standart) was loaded to the column and incubated overnight at room temperature. The resin was washed with thrombin-buffer and the target protein was harvested. Protein was concentrated by ultrafiltration (10 kDa MWCO, Amicon Ultra) up to 15 mg ml^−1^, centrifuged at 25,000×*g* for 30 min and supernatant loaded to a Superdex 75 pg (GE) Tricorn 10/300 gel filtration column equilibrated in buffer (20 mM HEPES, pH 8.0, 0.001% NaN3, 0.5 mM TCEP, 1 mM EDTA). Fractions with target protein were analyzed by SDS-PAGE.

### Cell culture and transfection

HEK293T cell line is commonly used in biological research and was purchased from ATCC. All cells were cultured at 37 °C in 5% CO2. HEK293T cells were cultured in Dulbecco’s modified Eagle’s medium plus 10% fetal calf serum supplemented with penicillin–streptomycin.

### Immunoprecipitaton and western blot analysis

HEK293 cells were transfected with DNA encoding for HA-p75 and Flag-TRAF6. NGF was added for 10 min and the cells were then spun down and lysed in 1 ml of lysis buffer (1% Triton X-100, 20 mM Tris, pH 8.0, 200 mM NaCl, 1 mM EDTA, 25 μg ml^−1^ phenylmethylsulfonyl fluoride and protease inhibitor cocktail). Lysates were then incubated with α-FLAG (SIGMA) overnight and with Sepharose-protein A for 2 h at 4 °C. The beads were washed and boiled with SDS-PAGE sample buffer and were subjected to Western analysis with the indicated antibodies.

### Experiment on p75-ΔECD and p75-ΔECD-T249C oligomerization

#### In bicelles

The rP75-ΔECD-3CX and rP75-ΔECD-3CX-249C were produced according to the previously described protocol^20^, but lauroylsarcosine was substituted to 50 mM sodium cholate during the IMAC. DMPC lipids were added to purified protein with 200 and 1,000 times excess and cholate was removed by dialysis against the bicelles-buffer (20 mM Tris, pH 8.0, 50 mM NaCl, 0.005% NaN3, 1 mM EDTA) with 20 mM bMe. To obtain bicelles samples, the CHAPS was added to the liposomes, and q = 1 DMPC/CHAPS bicelles were thus prepared. Finally, to remove reducing agent (bMe) the mixture was dialyzed against the bicelles-buffer with 3 mM CHAPS for 4–8 h with a high excess of buffer (> 1,000). The bicelles samples were incubated at room temperature and aliquots were analyzed by SDS-PAGE at several time points.

#### Kinetics

The formation of the covalent dimer in the bicelles system for both proteins (rP75-ΔECD-3CX and rP75-ΔECD-3CX-249C) can be described by the simplest irreversible reaction:

$$M+M \to D$$

where M and D denote monomer and dimer, respectively. The rate of loss of monomer can be written in differential form as:

$$\frac{d\left[M\right]}{dt} = -k{[M]}^{2}$$

where k is a second-order rate constant. The solution of this equation is

$${[M]}_{t}=\frac{{[M]}_{0}}{1+{[M]}_{0}kt}$$

where [M]_0_ is the initial concentration of monomers. Here and above all concentrations of proteins in liposomes are given in mole/mole units with respect to the concentration of lipid.

#### In the membrane of E. coli

For the production of rP75-ΔECD-3CX and rP75-ΔECD-3CX-249C *E. coli* strains were grown overnight in LB medium at 28 °C in a shaking incubator (New Brunswick Innova 44R) at 250 rpm. The OD600 was measured, equal amounts of cells were harvested by centrifugation at 7,000×*g* for 10 min and resuspended in 200 ml of M9 medium to resulting OD600 of 0.02. The cells were grown at 28 °C and 250 rpm. An equal amount of cells (normalized to OD600) was harvested by centrifugation after 2, 4, 6, 8, 24, 48 and 72 h of cellular growth after reaching an OD600 of 0.6 (~ 11 h after resuspension in M9). Cells were immediately resuspended in lysis buffer (10 mM Tris, 100 mM NaCl, 20 mM Iodoacetamide) and sonicated on ice in the dark. The lysate was clarified by centrifugation at 14,000×*g* for 30 min at 4 °C and membrane fraction (MF) with target protein was precipitated by centrifugation at ~ 200,000×*g* for 1 h at 4 °C. MF was resuspended in TES-buffer (10 mM Tris, pH 8.0, 5 mM EDTA, 1.5% SDS) with 20 mM iodoacetamide and analyzed by SDS-PAGE. The gel was stained using InVision His-Tag In-Gel Stain (Invitrogen) and analyzed using the ChemiDoc MP imaging system (BioRad) with Image Lab Software (BioRad).

The LPR value for bacteria is:

$$LPR=\frac{{N}_{lip}}{{N}_{prot}}$$

where N_lip_ and N_prot_ are the number of lipid and protein molecules for a single cell. The N_lip_ can be calculated as

$${N}_{lip}=\frac{2\times {S}_{cell}}{{S}_{lip}}$$

where S_cell_ and S_lip_ are a surface area of cell and “average” lipid, respectively. The N_prot_ can be calculated as

$${N}_{prot}=\frac{{C}_{prot}\times {N}_{A}}{MW\times {C}_{cell}}$$

where C_prot_ is an expression level of the protein, N_A_ is the Avogadro constant, MW is a molecular mass of the protein, and C_cell_ is a concentration of cells. To estimate the approximate LPR value in membrane of *E. coli* we used S_cell_ ~ 4 μm^2^^54^, S_lip_ ~ 0.6 × 10^–6^ μm^2^ (based on surface area of DMPC lipid^55^), N_A_ = 6 × 10^23^ mol^−1^, C_cell_ ~ 24 × 10^8^ cells ml^−1^ (based on OD600 of cell culture), MW = 21,486 g mol^−1^, C_prot_ ~ 6 mg l^−1^ (based on SDS-page analysis).

### NMR sample preparation

#### LPNs and liposomes assembly

The LPNs assembly was run according to the previously described protocol^20^. The dimyristoylphosphatidylcholine (DMPC) lipids and belt protein (MSP1D1) were added to the target protein after size-exclusion chromatography. To avoid the presence of several molecules (monomers or dimers) inside one LPN, high LPR ratio and large excess of MSP1D1 were used during the assembly. The BioBeads SM2 resin (BioRad) was added to the solution and detergent removing was controlled by ^1^H-NMR. To separate the devoid of p75 LPNs from the protein-bearing particles the metal affinity chromatography was applied in aqueous buffer (20 mM Tris, pH 8.0, 50 mM NaCl). The LPNs with target protein was eluted with 500 mM imidazole, dialyzed against 20 mM NaPi 6.5 and concentrated up to 0.4 mM by ultrafiltration with 30 kDa centrifugal filter units.

To prepare the protein incorporated into liposomes, DMPC lipids were added to the purified protein at LPR 150 and the mixture was dialyzed against 20 mM Tris, pH 8.0, 50 mM NaCl, 0.01% NaN3 until no detergent was detected in solution by ^1^H-NMR.

#### Deamidation experiments

To obtain the deamidated form of hP75DD the purified protein was concentrated up to 1 mM, dialyzed against the 50 mM NaPi pH 7, 0.5 mM TCEP, 1 mM EDTA, 0.01% NaN3 overnight and incubated at 30 °C for 2–3 weeks. To measure the kinetics of hP75DD deamidation, the samples in phosphate and HEPES buffers were analyzed by ^15^N-HSQC experiment at several time points.

### NMR spectroscopy

All NMR spectra were recorded using the Bruker Avance III 600 and 800 MHz spectrometers, both equipped with the cryogenic triple resonance probes. All the experiments were run at 30 C. To obtain the data about the structure and dynamics of rP75-ΔECD-3CX, we used the chemical shift index as an indicator of secondary structure and the NMR relaxation analysis as an indicator of disorder and backbone mobility. Standard set of triple-resonance spectra was used for the resonance assignment of rP75-ΔECD-3CX and p75-TMD-249C^56^. BEST-TROSY variants of pulse sequences were applied^57^ and non-uniform sampling was used to optimize the acquisition time^58^. Rotational diffusion was assessed based on the cross-correlated relaxation rates of amide groups as described in^29^. Translational diffusion was estimated using the PGSTE-W5-watergate pulse sequence with convection compensation^59^. Methyl group region was used to measure the diffusion coefficients to avoid the contribution of solvent exchange of labile protons and to maximize the sensitivity. Secondary structure prediction was made based on NMR chemical shifts in TALOS-N software^28^. Populations of hP75DD states were measured from the set of ^15^N-HSQC experiments using the deconvolution of one-dimensional projection of the cross-peaks from the NH group of Q369. Effective molecular weights of the proteins under investigation were assessed based on the hydrodynamic radii, using the relationship from the work^22^.

## Supplementary information

Supplementary information.
