# Supplementary material for: Revising the mechanism of p75NTR activation: intrinsically monomeric state of death domains invokes the "helper" hypothesis
Source: Sci Rep. 2020 Aug 13;10:13686. doi: 10.1038/s41598-020-70721-8 (PMC7427093; doi:10.1038/s41598-020-70721-8)
Supplement: Supplementary file 1 — Supplementary information. [file 41598_2020_70721_MOESM1_ESM.docx]

**Supplemental Information**

Revising the mechanism of p75NTR activation: intrinsically monomeric state of death domains invokes the "helper" hypothesis

Sergey A Goncharuk^1,+,*^, Liliya E Artemieva^1,2,+^, Kirill D Nadezhdin^1^, Alexander S Arseniev^1^, Konstantin S Mineev^1,*^

^1^ Laboratory of biomolecular NMR spectroscopy, Shemyakin-Ovchinnikov Institute of Bioorganic Chemistry of the Russian Academy of Sciences, Moscow, Russia.

^2^ Phystech school of biological and medical physics, Moscow Institute of Physics and Technology, Dolgoprudny, Russia.

^+^Goncharuk SA and Artemieva LE contributed equally to this work.

^*^ corresponding authors: Sergey Goncharuk [ms.goncharuk@gmail.com](mailto:ms.goncharuk@gmail.com); Konstantin Mineev [mineev@nmr.ru](mailto:mineev@nmr.ru)

***Table S1.*** *Hydrodynamic parameters of various p75 DD constructs*

| **construct/sample** | **τ_c_ ns** | **D_0_ ×10^-12^ m^2^/s** | **Predicted Mw^a^** | **Mw** |
| --- | --- | --- | --- | --- |
| *Rat DD His6^b^ (1 mM)* | 7.0±0.2 | 136±1 | 14.7/14.8 | 12.4 |
| *Rat DD (1 mM)* | 6.0±0.2 | 154±2 | 13.0/10.2 | 10.6 |
| *Rat DD C416S His6 (0.5 mM)* | 6.3±0.2 | 136±1 | 13.7/14.8 | 12.4 |
| *Rat DD C416S His6 80% cross-linked,* | 12.2±0.4 | 116±1^d^ | 26.5/24.2 | 24.8 |
| *Rat DD C416S (0.5 mM)* | 6.1±0.2 | 151±1 | 13.3/10.9 | 10.6 |
| *Rat DD C416S cross-linked* | 10.3±0.3 | 123±1 | 22.5/19.9 | 21.2 |
| *Rat DD monomer prediction for PDB 1NGR* | 5.8 | 145 | 12.7/12.3 | 10.6 |
| *Rat DD cross-linked dimer prediction for PDB 4F44* | 10.5 | 120 | 22.8/21.7 | 21.2 |
| *Human DD, NaP_i_, 1 mM* | 6.1±0.2 | 151±2 | 13.3/10.9 | 10.5 |
| *Human DD, HEPES, 1 mM* | n.a.^c^ | 158±2 | -/9.5 | 10.5 |
| *Human DD, D state, 1 mM* | 6.4±0.4 | n.a.^e^ | 14.1/- | 10.5 |
| *Human DD, isoD state, 1 mM* | 6.0±0.3 | n.a.^e^ | 13.0/- | 10.5 |
| *Human DD dimer prediction for PDB 2N97* | 19.6 | 98 | 42.7/38.6 | 21.2 |

*^a^Molecular weight was predicted based on the hydrodynamic radius of the equivalent sphere. The first number is estimated based on rotational diffusion / second number is obtained from the D_0_ values.*

*^b^His6 is the sequence MHHHHHHGSGSGLVPRGS at the N-terminus of the protein*

*^c^τ_c_ was not measured in HEPES, because the pattern of chemical shift splitting was identical to observed in NaPi*

*^d^The value is a weighted average between the 80% dimeric and 20% monomeric states*

*^e^Translational diffusion cannot be measured for the two states separately*


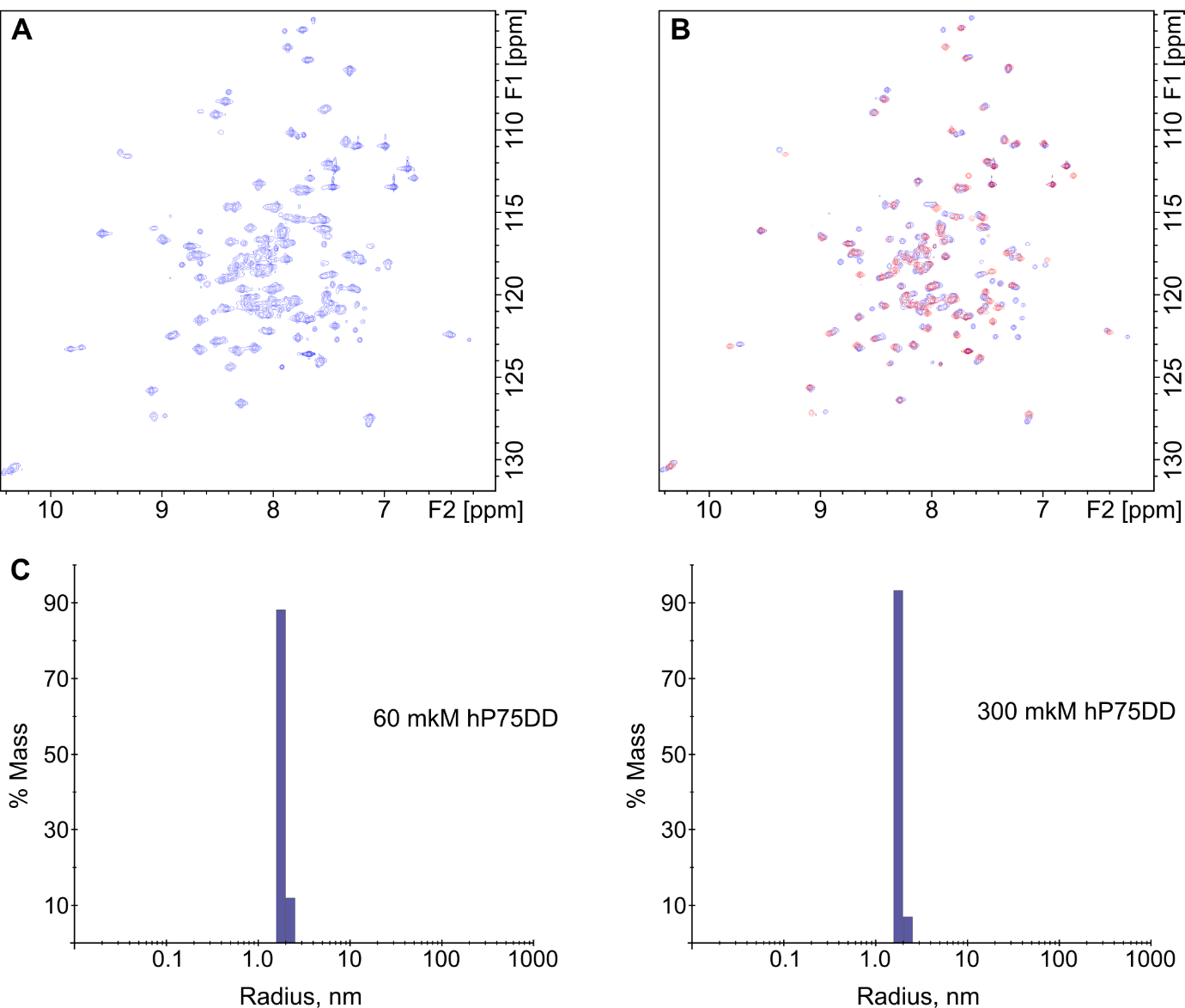


***Fig S1.*** *Analysis of hP75DD by NMR and DLS.* ***A.*** *^1^H,^15^N-HSQC NMR spectra of hP75DD in phosphate buffer 3 days after the NMR sample preparation.* ***B.*** *Overlay of two ^1^H,^15^N-HSQC NMR spectra of hP75DD at the initial state (immediately after protein purification and concentration) (red ) and after 19 days of protein incubation at 30 °C in 50 mM NaPi (blue).* ***C.*** *Distribution of the hydrodynamic radius of hP75DD measured by DLS at 60 mM and 300 mM of protein concentration in 50 mM NaPi solution pH 7.0, 30* *°C. The Figure was prepared using the program Inkscape 0.92 (https://inkscape.org/).*

*
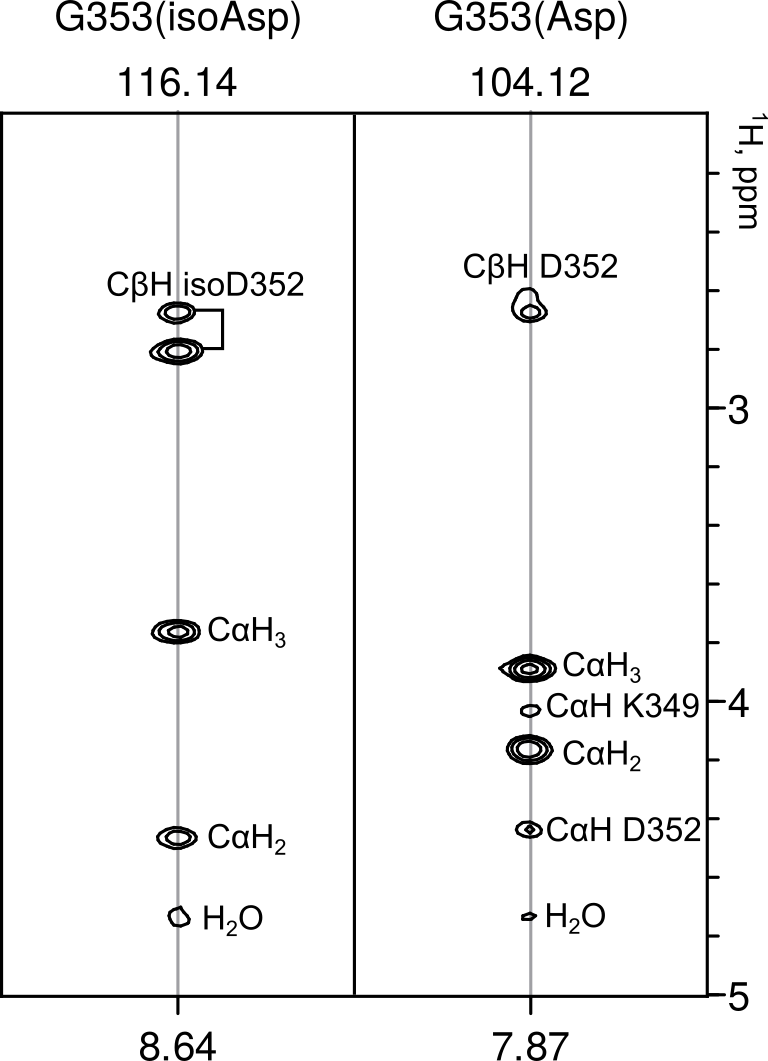
*

***Fig S2.*** *Two-dimensional strips of 3D-^1^H,^15^N-NOESY-HSQC spectra of hP75DD in 50 mM phosphate buffer after 95% deamidation. Strips correspond to the amide group signals of G353 in isoAsp352 and Asp352 forms of the modified proteins. The assignment of cross-peaks is provided, H_2_O corresponds to the exchange peak of the solvent. The Figure was prepared using the program Inkscape 0.92 (https://inkscape.org/).*


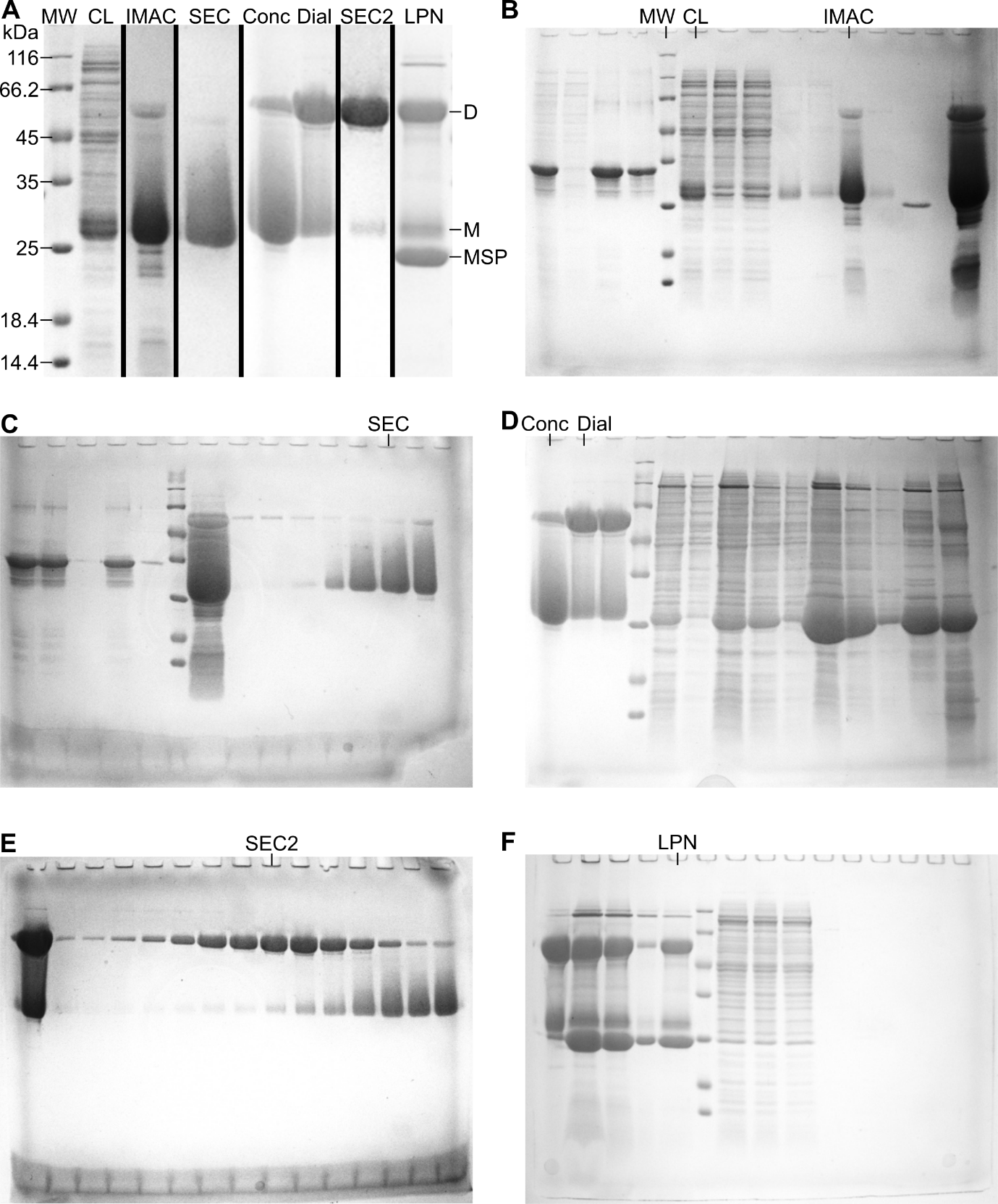


***Fig S3.*** *The rP75-ΔECD-3CX sample preparation. SDS–PAGE analysis in 14% gel, Tris-glycine buffer.* ***A.*** *MW - molecular weight marker, CL - total cell lysate, IMAC - protein purified by immobilized metal affinity chromatography (IMAC), SEC - protein purified by size exclusion chromatography, Conc - concentrated protein after 2 days of incubation at room temperature, Dial - concentrated protein after 2 days of dialysis at room temperature (detergent removing), SEC2 - dimer of rP75-ΔECD-3CX purified by size exclusion chromatography, LPN - protein incorporated into lipid-protein nanodiscs and purified by IMAC, D - dimer of rP75-ΔECD-3CX, M - monomer of rP75-ΔECD-3CX, MSP - the belt protein used for the nanodiscs assembly.* ***B-F.*** *The full-sized gels for A. The lines presented on A are identically signed. The Figure was prepared using the program Inkscape 0.92 (https://inkscape.org/).*

*
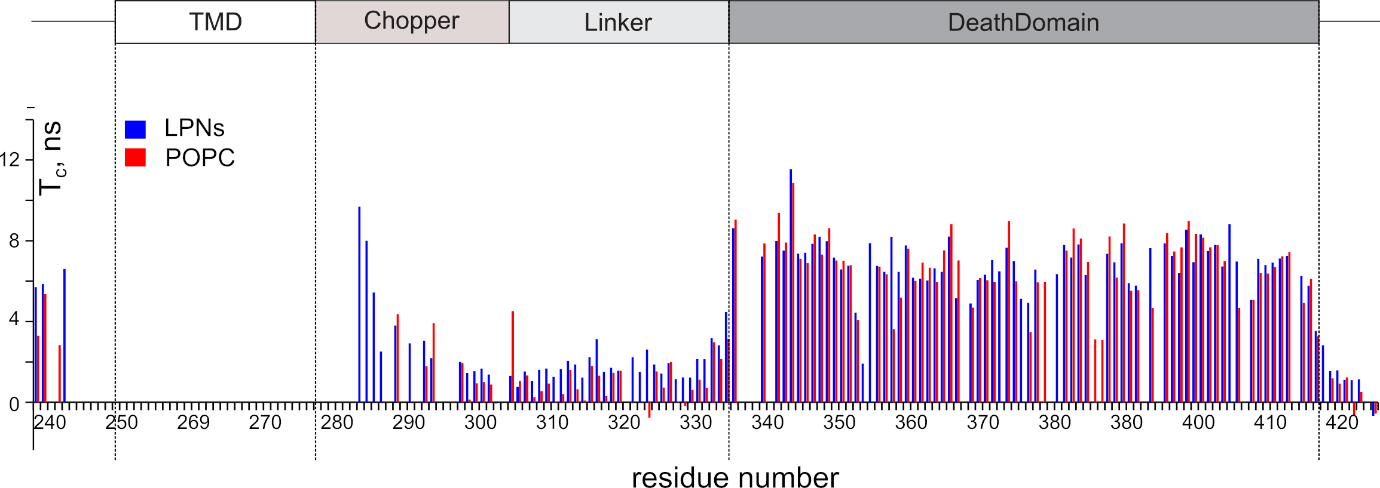
*

***Fig S4****. NMR-derived rotational diffusion correlation time of N-H bonds is plotted versus the residue number of rP75-ΔECD-3CX dimer in nanodiscs (blue bars) and POPC liposomes (red bars). The Figure was prepared using the program Inkscape 0.92 (https://inkscape.org/).*


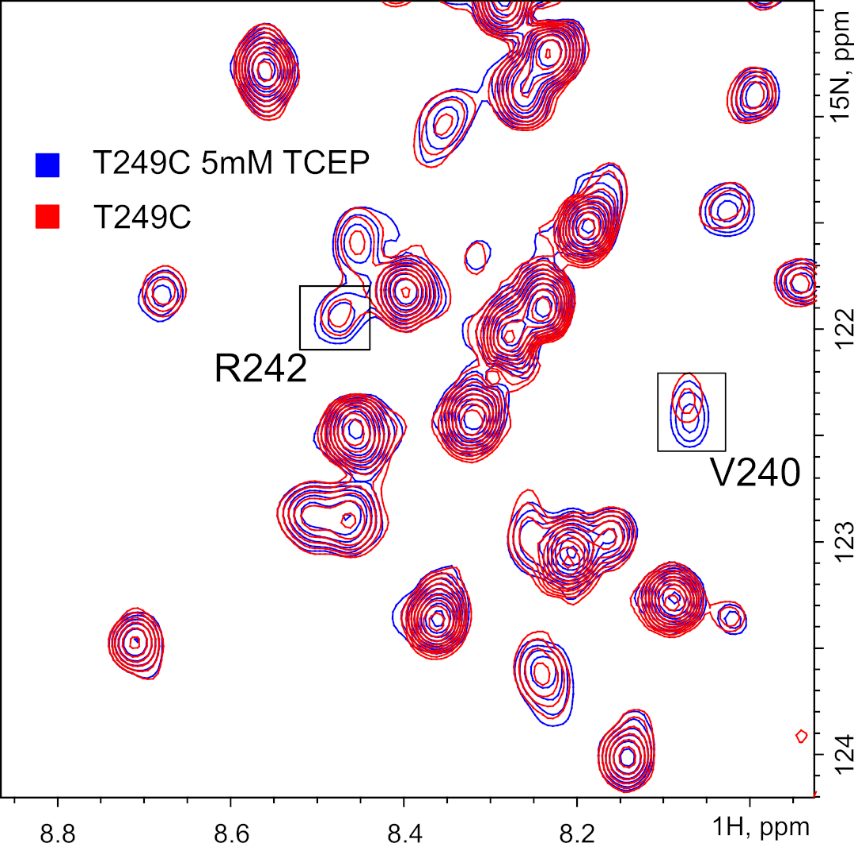


***Fig S5.*** *Fragment of 2D-^1^H,^15^N-TROSY-HSQC spectrum of dimeric rP75-ΔECD-3CX-T249C in MSP1D1 nanodiscs after (shown in blue) and before the addition of 5 mM TCEP (red). Signals with the most pronounced changes are indicated. The Figure was prepared using the program Inkscape 0.92 (https://inkscape.org/).*

*
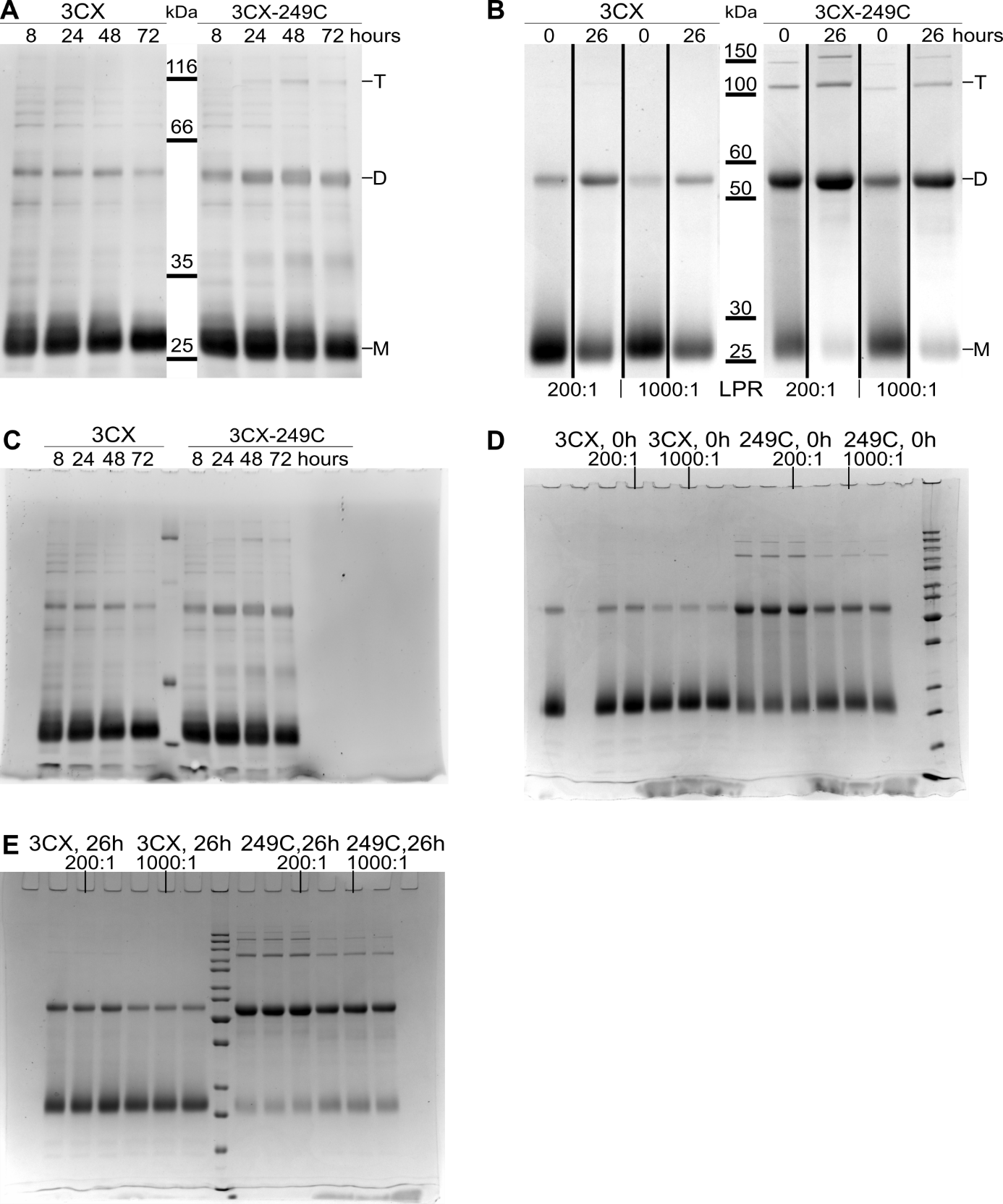
*

***Fig S6.*** *Comparison of rP75-ΔECD-3CX and rP75-ΔECD-3CX-249C oligomerization rates.* ***A.*** *Oligomerization in the membrane of E. coli BL21(DE3) cells. The non-reducing SDS-page analysis of E. coli membrane fraction through 8, 24, 48 and 72 hours after log-phase of the bacterial growth cycle (OD600 = 0.6). The cells' cultivations were under identical conditions for both strains. To avoid a disulfide bond formation during the sample preparation the iodoacetamide was added to all buffers. The gel was stained using InVision His-tag.* ***B.*** *Oligomerization in the DMPC:CHAPS bicelles at different LPR - 200:1 and 1000:1. 0 hours corresponds to the first sample preparation after removal of bME by dialysis.* ***C.*** *The full-sized gel for A.* ***D-E.*** *The full-sized gels for B. The lines presented on B are idenically signed. The Figure was prepared using the program Inkscape 0.92 (https://inkscape.org/).*


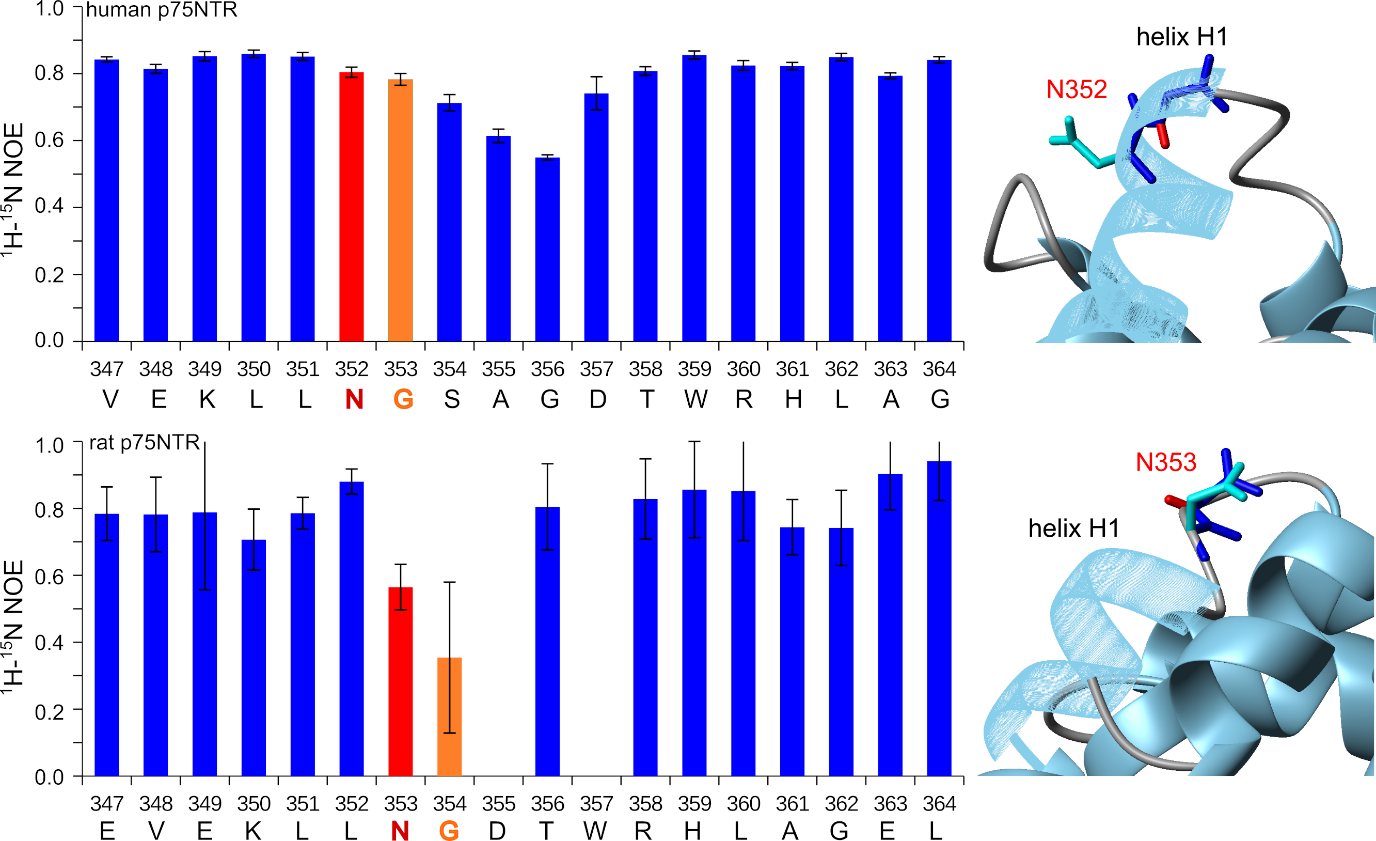


***Fig S7.*** *Dynamic parameters and conformation of N352G of hP75DD (top) and N353G of rP75DD (bottom). Heteronuclear steady-state NOE values and local conformation of NG fragment are shown for deamidated Asn and several adjacent residues of human and rat proteins. Asn is in the flexible loop in rat DD and in the stable region of Helix1 in the human protein. Two NOE values are not shown for rP75DD: D355 and W357 are too broad to measure the NOE reliably. Errors were calculated based on the spectral noise level. The Figure was prepared using the program Inkscape 0.92 (https://inkscape.org/).*

***
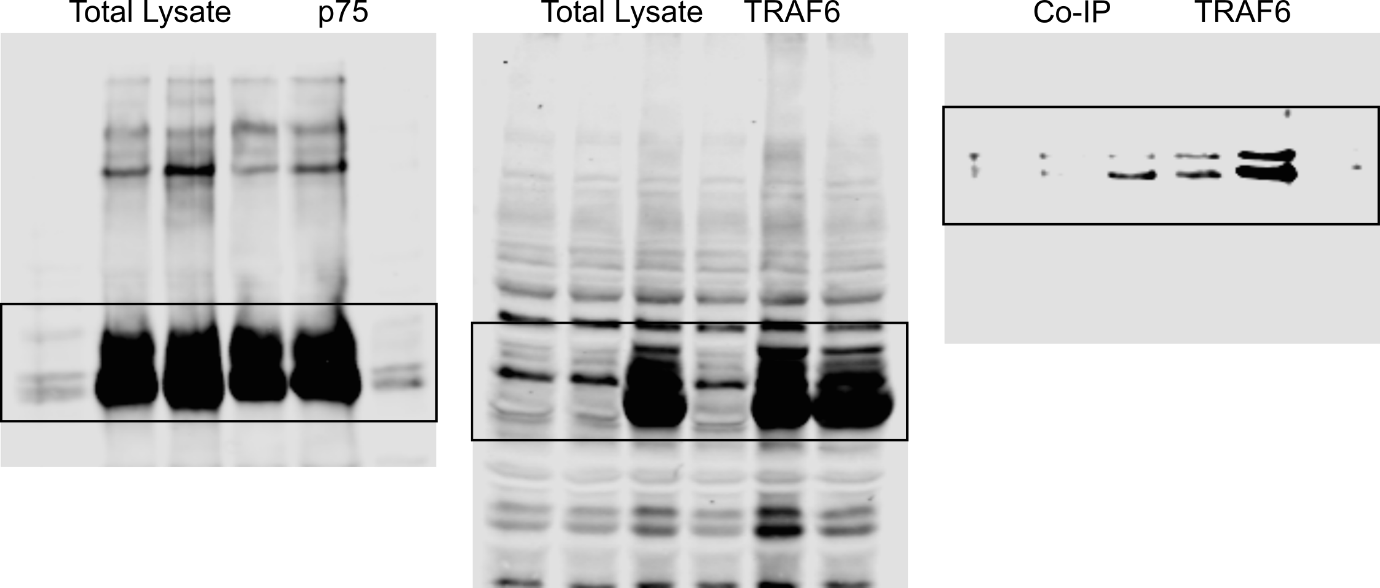
***

***Fig. S8.*** *The full-sized blots for Fig. 4. The cut parts are shown by rectangles. The Figure was prepared using the program Inkscape 0.92 (https://inkscape.org/).*

**
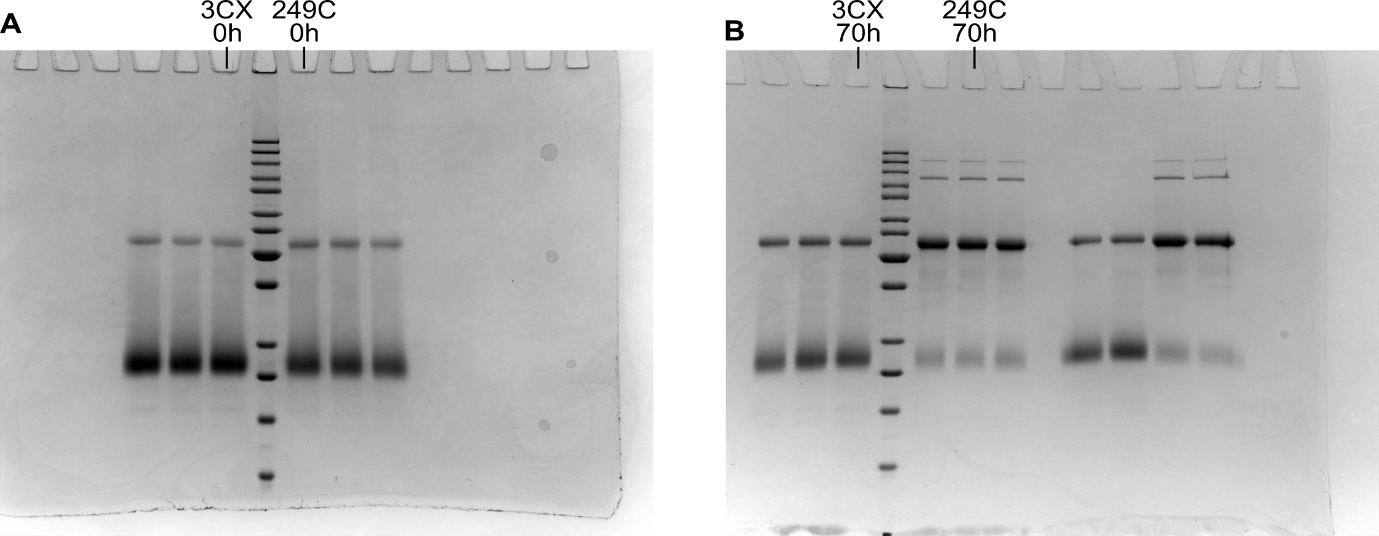
**

***Fig. S9.*** *The full-sized gel for Fig. 7. The lines presented on Fig. 7 are identically signed. The Figure was prepared using the program Inkscape 0.92 (https://inkscape.org/).*

**
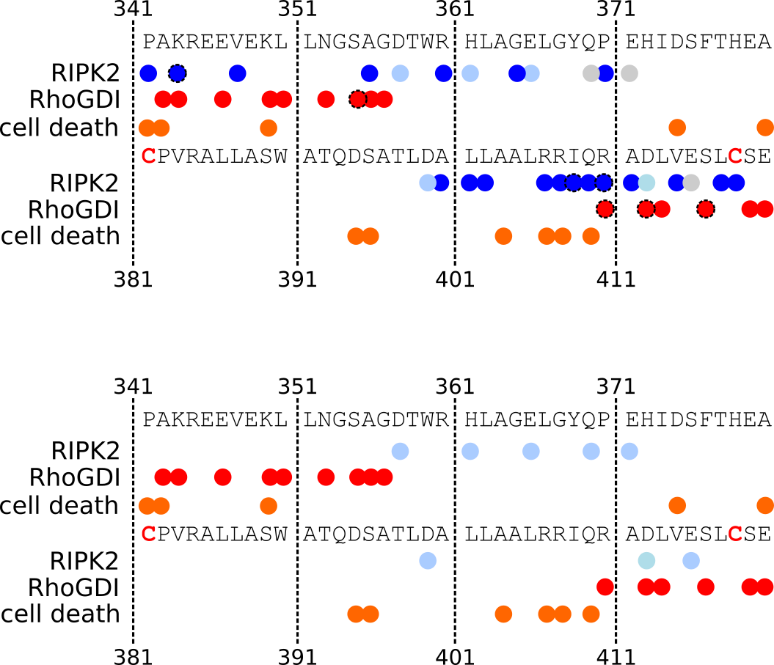
**

***Fig. S10.*** *Key residues of p75NTR DD, involved in the interactions with the adapter proteins RIPK2 (blue, according to PDB ID 2N80) and RhoGDI (red, according to PDB ID 2N83) and residues involved in the activations of JNK/caspase-3 and cell death (orange, according to the scanning mutagenesis ^1^. Residues, taking part in the intermolecular hydrogen bonding and salt bridges are highlighted with black circles. Residues of RIPK2, revealed by scanning mutagenesis are shown by gray circles* ^1^*. The Figure was prepared using the program Inkscape 0.92 (https://inkscape.org/).*

References.

1. Charalampopoulos, I. *et al.* Genetic Dissection of Neurotrophin Signaling through the p75 Neurotrophin Receptor. *Cell Reports* **2**, 1563–1570 (2012).
